# Prolactin drives cortical neuron maturation and dendritic development during murine embryonic stem cell differentiation

**DOI:** 10.3389/fcell.2025.1551090

**Published:** 2025-02-26

**Authors:** Omar Martinez-Alarcon, Daniela Colin-Lagos, Ximena Ramirez-Meza, Alejandra Castilla, Georgina Hernandez-Montes, Eliezer Flores-Garza, Alejandro Lopez-Saavedra, Daniela Avila-Gonzalez, Alejandro Martinez-Juarez, Anayansi Molina-Hernández, Nestor Emmanuel Diaz-Martinez, Wendy Portillo, Nestor Fabian Diaz

**Affiliations:** ^1^ Departamento de Fisiología y Desarrollo Celular, Instituto Nacional de Perinatología, Ciudad de México, Mexico; ^2^ Bioterio, Instituto de Neurobiologia, Universidad Nacional Autonoma de Mexico, Quéretaro, Mexico; ^3^ Red de Apoyo a la Investigación, Coordinación de la Investigación Científica (Universidad Nacional Autonoma de Mexico), Instituto Nacional de Ciencias Médicas y Nutrición, Mexico City, Mexico; ^4^ Department of Bioengineering, Imperial College London, London, United Kingdom; ^5^ Advanced Microscopy Aplications Unit (ADMiRA), Instituto Nacional de Cancerologia, Mexico City, Mexico; ^6^ Tecnologico de Monterrey, Escuela de Medicina y Ciencias de la Salud, Monterrey, Mexico; ^7^ Laboratorio de Reprogramación Celular y Bioingenería de Tejidos, Biotecnología Médica y Farmacéutica, Centro de Investigación y Asistencia en Tecnología y Diseño del Estado de Jalisco, Guadalajara, Mexico; ^8^ Departamento de Neurobiología Conductual y Cognitiva, Instituto de Neurobiología, Universidad Nacional Autonoma de Mexico, Quéretaro, Mexico

**Keywords:** prolactin, prolactin receptor, cortical neurons, neural stem cells, pluripotent stem cells

## Abstract

**Introduction:**

Prolactin (PRL) is a pleiotropic hormone implicated in various physiological processes; however, its contribution to neurodevelopment, particularly early corticogenesis, remains insufficiently characterized. In this study, we investigate PRL’s regulatory influence on the initial stages of cortical development, with an emphasis on its effects on neuronal and astrocytic differentiation.

**Methods:**

We employed a standardized in vitro differentiation protocol to generate cortical neurons from mouse embryonic stem cells (mESCs). Prolactin receptor (PRLr) expression was evaluated in pluripotent stem cells, neural stem cells (NSCs), immature neurons, and mature neurons using both PCR and immunofluorescence. These analyses revealed dynamic changes in PRLr expression throughout the differentiation process. Additionally, cells were treated with varying concentrations of PRL during early and late differentiation phases, enabling assessment of its impact on neuronal phenotypic distribution and morphological complexity.

**Results:**

Early PRL administration significantly enhanced the population of β-tubulin III + immature neurons, promoting neuronal survival without altering NSC proliferation. Furthermore, PRL treatment increased the abundance of Tbr1 + and NeuN + neurons, augmented dendritic complexity, and accelerated neuronal maturation. In contrast, PRL exposure at later stages of neural differentiation did not yield comparable effects. Notably, PRL delayed the maturation of protoplasmic astrocytes, although the total astrocyte population was not affected.

**Discussion:**

These findings highlight PRL’s pivotal role as a regulator of early corticogenesis by modulating neuronal survival, dendritic development, and astrocyte maturation. PRL thus emerges as a potential key factor in neurodevelopment, underscoring its importance in the hormonal regulation of neural differentiation and maturation. These insights may have broader implications for understanding the molecular and cellular mechanisms underlying normal and pathological neurodevelopment.

## Introduction

Prolactin (PRL) is a hormone involved in over 300 physiological processes, extensively studied in contexts such as lactation and reproduction in mammals (Bole-Feysot et al., 1998). Despite its well-established roles in adult animals, its potential involvement in early development remains poorly understood. The hormone is synthetized in a variety of tissues other than the adenohypophysis during the life cycle of individuals. For example, it has been demonstrated to play a vital role in the placenta and is necessary for the implantation of fertilized eggs in humans and the maintenance of corpus luteum during pregnancy in rodents (Martínez-Alarcón et al., 2022). Additionally, the PRL receptor (PRLr) is expressed in various tissues and developmental stages, including early stages of mouse development (Bole-Feysot et al., 1998; Martínez-Alarcón et al., 2022; Vlahos et al., 2001).

PRLr is a transmembrane receptor in the cytokine receptor family that, upon ligand binding, activates multiple intracellular signaling pathways. The most well-characterized among these are the Janus kinase/signal transducer and activator of transcription (Jak-Stat), phosphoinositide 3-kinase (Pi3k)/Akt and mitogen-activated protein kinase (Mapk) pathways (Chasseloup et al., 2024). These cascades regulate essential cellular processes including survival, proliferation, differentiation and gene expression.

Emerging evidence from human and animal studies suggests that dysregulation of PRL and PRLr signaling may affect neurodevelopment. Notably, altered PRL levels have been observed in conditions such as preeclampsia, anencephaly, and intrauterine growth restriction (Arosio et al., 1995; Thorpe-Beeston et al., 1992), all of which coincide with critical periods of neuronal migration, synaptogenesis and gliogenesis during fetal brain development.

Indeed, the PRLr participate in adult neurogenesis and behavior regulation (Larsen and Grattan, 2012), maternal neurogenesis and *postpartum* behavior regulation (Larsen and Grattan, 2012; Larsen et al., 2008; Mak et al., 2007; Shingo et al., 2003). PRL promotes the proliferation and differentiation of neural stem cells (NSCs) into neurons and glia from human fetal cortex as well as the adult mouse hippocampus and subventricular zone (Larsen and Grattan, 2012; Pathipati et al., 2011). Interestingly, during development, PRL concentrations are significantly higher in amniotic fluid compared to maternal blood, suggesting it acts as a signal to neuroepithelial cells and NSCs during neural tube closure (Luciano and Varner, 1984; Schenker et al., 1975; Winters et al., 1975). Despite these insights, the direct role of PRL in early neurogenesis remains poorly understood.

The differentiation of mouse embryonic stem cells (mESCs) into cortical neurons provides a robust *in vitro* model for early corticogenesis (Gaspard et al., 2009; Gaspard et al., 2008). This protocol recapitulates the progression from pluripotency to the formation of mature cortical neurons and glial cells, offering a powerful system to investigate the molecular and cellular roles of PRL and PRLr during neurodevelopment. While previous studies have underscored the general importance of PRL in neurodevelopment, its specific function in regulating cortical neuron maturation and astrocyte differentiation remains underexplored.

Here, we investigate the role of PRL in cortical neuron differentiation from mESCs. By examining the dynamics of PRLr expression, PRL signaling and their influence on both neuronal and astrocytic lineage commitment, this study aims to elucidate the regulatory mechanisms underlying PRL’s involvement in early corticogenesis.

## Methodology

### Animals

Mice were handled according to the National Institute of Health’s Guide for the Care and Use of Laboratory Animals and the Institutional Committee on Animal Care and Use of Laboratory Animals of the Institute of Neurobiology, UNAM. Briefly, C57BL/6J were mated and pregnancy was confirmed by a vaginal plug corresponding to embryonic day 0 (E0). The pregnant mice were housed individually under a 12 h/12 h light/dark artificial cycle with controlled temperature, ad food (LabDiet 5001) and water *ad libitum*.

### Embryo dissection

Mouse embryos at stages E12.5 to E14.5 were harvested from the uterine horn and dissected in PBS 1X on ice. The embryos were fixed in 4% paraformaldehyde (PFA) for 4 h at room temperature, then preserved in PBS 1X. Dissections were performed under a stereomicroscope using microsurgical tools, and the embryos were kept in PBS 1X until further processing.

### Light-sheet microscopy tissue processing

Embryos were cleared using previously published protocol, ScaleS (Hama et al., 2015). Briefly, the embryos were incubated in a series of solutions (S0, S1, S2, S3, and S4) with increasing concentrations of urea (Meyer), sorbitol (Sigma- Aldrich), and other clearing compounds (DMSO, glycerol, Triton X-100 all from Sigma Aldrich). Each step was carried out at 37°C for 12 h. Once cleared, the tissues were stored in Scale S4 at 4°C until immunohistochemistry.

For immunostaining, the tissues were incubated in PBS for 6 h at room temperature. Primary antibodies diluted in AbScale Solution (PBS 1X, 0.33M urea, 0.3% Triton X-100) were incubated for 48 h at 37°C, followed by two 2-h washes at room temperature in AbScale solution. Secondary antibodies (Supplementary Table S3) were applied under the same conditions as the primary antibodies. After secondary antibody incubation, the tissues were rinsed for 6 h with AbScale solution and then were rinsed twice for 2 h at room temperature in antibody rinse solution (PBS 1X, 2.5% BSA, and 0.05% Tween-20). Afterward, tissues were fixed again in 4% PFA for 1 h, washed with PBS for 1 h, and stained with *6-diamino-2-phenylindole (DAPI)* (Invitrogen) for 20 min at room temperature. Finally, the tissues were washed twice in Scale S4 (without sorbitol) for 10 min each and stored at 4°C in Scale S4 until imaging.

### Mounting and visualization in light sheet microscope

The cleared tissues were embedded in 2% low-melting-point agarose (Invitrogen) dissolved in 1X PBS. The samples were aligned upright, with the anterior-posterior axis positioned appropriately in the glass capillary. For imaging, the agarose was pushed out, suspending the tissue in a chamber containing Scale S4 (without sorbitol) as the mounting medium. The refractive index (RI) of the Scale S4 solution was measured with a refractometer (RI = 1.43) and the microscope lens was adjusted accordingly. All images were acquired using the ZEISS Lightsheet Z.1 Lightsheet Fluorescence Microscope (Carl Zeiss, AG), RRID:SCR-020919, with illumination objective: LSFM clearing 10x/0.2 and detection objective: LSFM clearing 20x/1.0 Corr (n = 1.43) (filter used BP 420-470, BP 505-545 and BP 575-615). Detection settings (30 ms of exposure) were applied for optimal visualization.

### Cell culture

#### Feeder layer

Mouse Embryonic Fibroblasts (MEFs) were obtained from day 13.5 embryos. To this end, pregnant CD-1 females were sacrificed by cervical dislocation at gestational day 13.5. Embryos were collected and the heads and red tissues were discarded, while the remaining tissues were enzymatically dissociated. After inactivating the enzymatic process, the cell suspension was cultured in DMEM (Dulbecco’s Modified Eagle’s medium; Gibco, United States) with 10% fetal bovine serum (Gibco). Once the cell culture reached full confluence, MEFs were either cryopreserved or subculture for inactivation by mitomycin C (Sigma-Aldrich, United States) (10 μg/mL) for 3 h at 37°C. Afterward, the cells were trypsinized, resuspended in MEF medium and cultured at a density of 5 × 10^4^ cells/cm^2^ for use as feeder layers.

#### Mouse embryonic stem cells culture

Mouse embryonic stem cells (ES-R1, ATCC-1011) (Nagy et al., 1993) were stored in liquid nitrogen and thawed in a 37°C water bath before being cultured. Cells were maintained in an undifferentiated state on gelatin-coated culture plates using ES medium [DMEM (Dulbecco’s Modified Eagle’s medium) (Gibco, United States) supplemented with 15% fetal bovine serum, 1,000 U/mL LIF (Millipore, USA), 0.1 mM non-essential amino acids (Gibco), 1 mM sodium pyruvate, 50 U/mL penicillin/streptomycin (Gibco) and 0.1 mM 2-mercaptoethanol (Sigma-Aldrich)] at 37°C in a 5% CO_2_ incubator (Series II, Thermo Scientific, United States). The medium was changed daily.

Mycoplasma-free cultures were confirmed using the MycoFluor™ *Mycoplasma* Detection Kit (Thermo Scientific). Sterility was evaluated in antibiotic- and antimycotic-free medium through daily observations prior to the start of the experiments. Differentiation potential was assessed through embryoid body assays and default differentiation, resulting in cells positive for markers representative of the three germ layers, neural morphologies, and contractile cells. For all experiments, the undifferentiated state of the cells was validated by evaluating the core pluripotency markers through PCR and immunofluorescence.

#### *In vitro* differentiation of ESC to cortical neurons

We followed the protocol described previously in the literature (Gaspard et al., 2009; Gaspard et al., 2008). Briefly, mESC at 80% confluence were washed with PBS (Sigma) and detached using EDTA/trypsin. The feeder layer was removed by selective adhesion on a culture plate treated with gelatin, and the unattached mESC were seeded into a new culture plate in Default Differentiation Medium (DDM) (DMEM/F12 + GlutaMAX) supplemented with N2 (1x), 0.1 mM non-essential amino acids, 1 mM sodium pyruvate, 0.1 mM 2-mercaptoethanol, 50 U/mL penicillin/streptomycin (all from Gibco), and 500 μg/mL BSA (Biowest, France) at a density of 7.5 × 10^3^ cells/cm^2^ marking the initiation of early differentiation (Day 0–12), this phase is characterized by the transition of mESC from a pluripotent state to NSCs and early progenitors. The cultures were maintained at 37°C in a 5% CO_2_ environment.

On early differentiation day 2, the DDM was supplemented with 1 μM cyclopamine. The medium was changed every other day until early differentiation day 10, when the medium was replaced with cyclopamine-free DDM and incubated for an additional 2-day period. On day 12 of early differentiation, the cells were passaged using EDTA/trypsin and a single-cell suspension was ensured through gentle pipetting. A total of 1.25 × 10^5^ cells per cm^2^ were reseeded in culture dishes pre-treated with poly-L-ornithine/laminin in N2/B27 medium (DMEM plus N2 and B27, both from Gibco) at 37°C, 5% CO_2_. The medium was changed every 2 days until the end of the experiment (Day 21 and 28). The late differentiation phase (Day 13–28) is characterized by the transition of NSCs and progenitors into mature neurons and glial cells.

### Prolactin treatments

To determine the effects of PRL on differentiation of mESC to cortical neurons, cells were treated with different concentrations of the hormone either during the early differentiation period or during the late differentiation period. Murine PRL (PreproTech, New Jersey, USA) was reconstituted in PBS (Sigma) with 0.1% BSA. The stock hormone solution was aliquoted in working volumes, stored at −20°C and never refrozen once thawed. In a series of experiments, we performed a dose-response curve of the hormone with the following concentrations 0.2, 0.4, 2, 6, and 20 nM, encompassing physiological concentrations during the prenatal development (Pathipati et al., 2011).

To assess the hormone’s effect on cultures we administered PRL daily directly into the medium to achieve each of the final concentration. Control received only the medium, while another group (vehicle) received 0.1% BSA.

### RNA extraction and RT-PCR

Assays were performed as described (Avila-Gonzalez et al., 2022). Briefly, total RNA was isolated from the cells and tissues using TRIzol (Life Technologies). The purity of the RNA was assessed by 260/280 nm ratio (1.8-2.2) using a spectrophotometer (NanoDrop One, Thermo Scientific) and was further evaluated through 2% agarose gel electrophoresis.

RNA (2,000 ng) was reverse transcribed in a final volume of 20 μL containing 4 μL of 25 mM MgCl_2_, 2 μL of reverse transcriptase buffer, 2 μL of 10 mM dNTP mix, 0.5 μL of ribonuclease inhibitor, 0.2 μL of AMV reverse transcriptase enzyme (15 U) (Promega), 0.2 μL of primers, and nuclease-free water, for a final volume of 20 μL. The components were thoroughly mixed and incubated at 42°C for 20 min. The reaction was terminated by heating at 95°C for 5 min to inactivate the enzyme.

For cDNA amplification, each reaction was prepared with the following components: 4 μL of GoTaq 5X Flexi Buffer (Promega), 0.8 μL of 25 mM MgCl (Promega), 0.4 μL of 10 mM dNTPs (Promega), 0.2 μL of each primer (25 p.m.) (Supplementary Table S2), 0.2 μL of GoTaq DNA polymerase (Promega), and the necessary volume of nuclease-free water to reach a total volume of 10 μL.

The PCR was performed with the following cycling conditions: initial denaturation at 95°C for 10 min, followed by 35 cycles of denaturation at 95°C for 1 min, annealing for 1 min and extension at 72°C for 1 min. A final extension step was carried out at 72°C for 5 min.

PCR products were then analyzed on a 2% agarose gel and the size of the products were determined by comparison with a molecular weight standard after GelRed (Biotium) staining. Reactions using RNA without reverse transcription were included as a negative control for PCR amplification.

### Immunofluorescence

We followed the previously reported protocol (Avila-Gonzalez et al., 2022). In brief, cells and tissue were fixed with 4% PFA for 20 min, washed with PBS (Sigma), permeabilized with 0.3% Triton X-100 for 30 min, blocked with 5% BSA for 30 min and incubated with primary antibodies (Supplementary Table S2) in blocking solution overnight at 4°C.

The next day, cells were washed with PBS (Sigma) 1X and incubated with secondary antibodies (Supplementary Table S2) conjugated to fluorophores at a concentration of 1:1000 for 2 h at room temperature. A solution of DAPI (5 μg/mL) (Thermo-Fischer) was applied to stain the nuclei. As negative control, cells and tissues were incubated only with the secondary antibody (data not shown). After washing, cells were mounted.

### EdU assay

On day 14 of differentiation, the cells were incubated with 10 μM EdU for 1 h at 37°C. At the end of the incubation, the solution was removed, and the cells were washed twice with PBS (Sigma) and fixed with 4% PFA for 20 min. Following fixation, the cells were washed twice more with PBS (Sigma). To detect EdU-positive cells, they were permeabilized with 0.5% Triton-X100 for 20 min and then incubated with the reaction cocktail (1X Click-iT EdU reaction buffer, 4 mM CuSO_4_, 5 μM Alexa Fluor 488 azide and Click-iT EdU buffer additive; Thermo Fisher) according to the provider’s instructions for 30 min. After, the cells were washed with PBS (Sigma) and incubated with DAPI at room temperature for 5 min. Finally, to determine the population of proliferative NSC, the cells were incubated with an anti-nestin antibody overnight at 4°C, followed by incubation with a secondary antibody conjugated to a fluorophore for 2 h at room temperature, as previously described.

### Cell counting

Cell counts from immunofluorescence experiments were performed using microphotographs taken with an epifluorescence microscopy (Olympus IX81) equipped with a CCD camera (Hamamatsu ORCA-Flash 2.8, Japan). The analysis of cultures was conducted by counting the number of cells expressing the marker of interest in nine random fields at ×20 magnification, in duplicate, from three to five independent experiments. The number of positive cells for each marker analyzed was determined using ImageJ software.

Another series of images was acquired using a confocal microscope (Zeiss AX10) with a ×20 objective to detect Alexa 488 and 568 fluorescence sequentially, by excitation with different lasers. The confocal settings were adjusted to minimize bleed-through between channels. Subsequently, the images underwent processing with Zen blue software and the analysis was carried out using the Fiji software. Quantification of fluorescence intensity was also performed with Fiji, ensuring consistent threshold settings across all images to maintain accuracy.

For the merge plot, the images obtained through confocal microscopy were used to quantify intensity values along a diagonal axis of 390 μm, allowing the generation of intensity profiles for the three fluorescence channels used in image acquisition. All values were obtained in triplicate from different images, then averaged and plotted in temporal order according to the day they were obtained. This analysis was based on the methodology described by Weberling and Zernicka-Goetz (2021).

### Bioinformatic analysis

Aligned reads were obtained from publicly available data sources, specifically from Encyclopedia of DNA Elements (ENCODE) (Gorkin et al., 2020; He et al., 2020). From the various raw and processed data types available, pre-aligned reads, which had been aligned to a reference genome to identify and name transcripts, were selected. Focus was placed on reads corresponding to forebrain (FB) tissues, and both sets of duplicated reads were downloaded to ensure greater statistical robustness.

Data preprocessing involved filtering out reads not named using the ENSEMBL nomenclature and those with a CPM (counts per million) less than 0.5. Differential gene expression analysis was conducted using the DESeq2 tool (1.36.0) (Love et al., 2014) with an FDR (false discovery rate) cutoff of 0.1 and a minimum fold change of 2 to investigate gene expression changes between tissues and within the same tissue at different time points.

The differentially expressed genes (DEGs) identified were further analyzed to determine enriched pathways between comparisons using the GAGE method (2.46.1) (Luo et al., 2009) with an FDR cutoff of 0.1. For the pathways of interest, graphical representations were generated using KEGG (Kanehisa et al., 2016). These representations included the list of genes within the pathways and their expression values under each experimental condition.

### Dendritic complexity and Sholl analyses

Dendritic complexity and Sholl analyses were performed on an average of 45 neurons at day 14 of differentiation in each group (Control, Vehicle and PRL), excluding neurons with aberrant morphology or truncated dendrites. Two-dimensional dendritic arbors were drawn using Samsung Notes from the micrographs obtained at ×40 magnification on an optical microscope (Olympus IX-81, Japan). Dendritic profiles were analyzed using Fiji (ImageJ) software, applying a consistent scale across all images. For Sholl analysis (Sholl, 1953), a series of concentric circles with 5 μm spacing, centered on the soma was overlaid on each image. The number of intersections made by dendritic arbors with these circles was quantified using the Sholl analysis function in Fiji.

The total number of primary, secondary or tertiary dendrites was manually counted using Fiji. To calculate the dendritic complexity index (DCI), the following equation was employed (Chameau et al., 2009):

$$\text{DCI}=\frac{Ʃ\;ordinal\;value\;of\;the\;dendrite+\#\;of\;total\;dendrites}{\#\;of\;primary\;dendrites}x\;dendritic\;arbor\;length$$

The dendritic length was measured using the same Fiji software, ensuring that all measurements were consistent and accurate.

### Data analysis

Data are presented as a means ± standard error of the mean (SEM). The normality of the data was assessed using the Shapiro-Wilk normality test. For comparison between control and treated groups, a one-way ANOVA followed by Tukey’s *post hoc* multiple comparison test was performed. To compare treatment effects during early differentiation versus late differentiation, an unpaired t-test was used, provided the data passed the Shapiro-Wilk normality test. P values < 0.05 were considered statistically significant. Statistical analyses and graph constructions were conducted using GraphPad Prism 8.0.1 (GraphPad Software, La Jolla, CA, United States).

## Results

### Presence of the prolactin receptor in mouse embryo development during early neurogenesis

**FIGURE 1:**
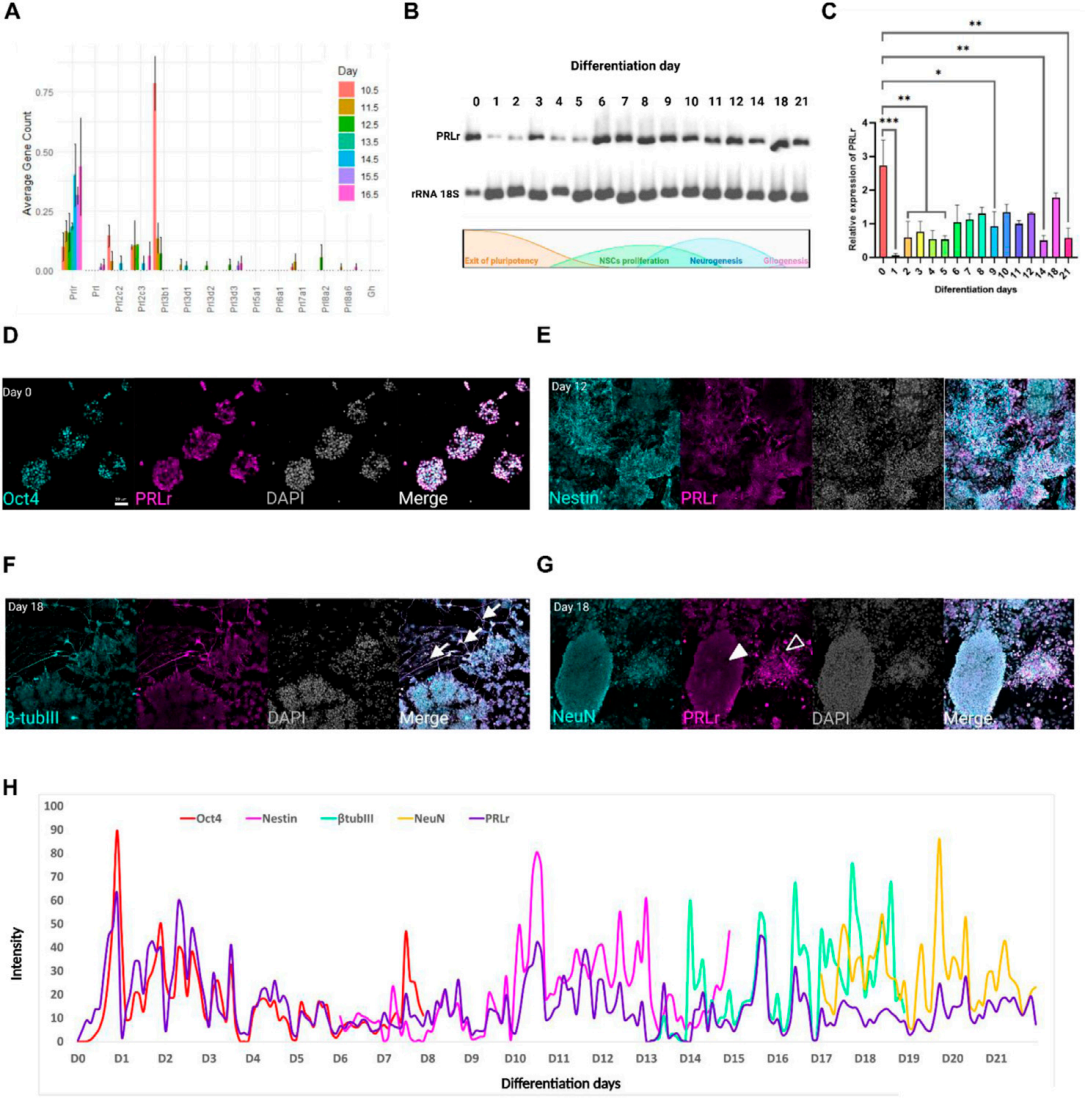
Dynamic PRLr expression during mESC differentiation into cortical neurons. **(A)** Expression profile of PRL and PRLr ligands, including placental lactogens and GH, in the forebrain (FB) from E10.5 to 16.5. **(B)** Representative electrophoresis gel showing PRLr expression analyzed via PCR from Day 0–21 during differentiation; ribosomal 18S RNA served as a loading control. A schematic of the differentiation protocol is included to link PRLr expression with key cellular events. **(C)** Quantitative analysis of PRLr relative expression over the differentiation course, presented as mean ± SEM (n = 3 biological replicates per day). Statistical analysis revealed significant differences between Day 0 and other time points as indicated by horizontal lines (*p < 0.05, **p < 0.01, ***p < 0.001). The single line across Day 2–5 illustrates grouped comparisons versus Day 0 for clarity. Statistical analysis was performed using one-way ANOVA with Sidak’s multiple comparisons test (F, DFn, Dfd values were: 3.899, 15 and 32). **(D–G)** Confocal micrographs of PRLr (magenta) co-localized with markers: Oct4 (pluripotency, Day 0), Nestin (NSC, Day 12), β-tubulin III (immature neurons, Day 18) and NeuN (mature neurons, Day 18) in cyan. Noteworthy features include PRLr-positive neurites in immature neurons (arrows) indicating areas of PRLr co-localization along β-tubulin III positive cells and NeuN-positive cells with low (filled arrowheads) or high PRLr signals (open arrowheads). Scale bar: 50 μm. **(H)** Merged plot showing PRLr intensity alongside Oct4, Nestin, β-tubulin III and NeuN markers from Day 0–21, marking transitions in neural differentiation. Intensity profiles analyzed using Fiji and Zen Lite software.

To explore the potential role of PRL and its receptor during early neurogenesis, we analyzed the expression levels of PRLr and PRL in the FB between embryonic day (E)10.5 to E16.5 at 24-h intervals. This analysis utilized data from a published mouse embryo transcriptome dataset in the ENCODE consortium (He et al., 2020), encompassing critical stages of neurogenesis and corticogenesis. Our results showed consistent PRLr transcript expression in the FB throughout this developmental period, with levels at E16.5 increasing to more than three times those observed at E10.5 (Figure 1A).

Growth hormone (GH), placental lactogens and PRL-like proteins are key regulators of embryonic growth, proliferation and differentiation, with overlap with PRL due through their interactions with common receptor. GH can function in an autocrine/paracrine manner before the hypophysis becomes fully established, thereby contributing to early developmental process. Placental lactogens similarly modulate fetal metabolism and tissue growth, often via insulin-like growth factors (IGF). Given the increased PRLr expression, we investigated its potential receptor ligands (Elkins et al., 2000; Handwerger and Freemark, 2000; Karabulut et al., 2001; Oberbauer, 2015; Somers et al., 1994). Using Peng He’s dataset, we examined genes from the PRL family annotated with the molecular function “prolactin receptor binding”. PRL was expressed at low levels and GH was absent; specifically, PRL expression was detected only at E15.5 and E16.5 (Figure 1A), consistent with prior reports indicating minimal embryonic PRL production, with maternal sources being the primary contributor (Martínez-Alarcón et al., 2022). Placental lactogens and PRL-like proteins showed varying expression patterns: Prl3b1 was highest at E10 and declined thereafter, while Prl2c2 and Prl2c3 remained relatively high through E14.5. Overall, these findings suggest that PRLr may be relevant in early neurogenesis through interactions with maternal PRL or PRL-like ligands, supported by the temporal increase in PRLr and the presence of specific placental lactogens, even in the absence of substantial endogenous PRL.

During this period, NSCs markers (Sox2, Nestin and Pax6) were expressed at levels roughly 10-fold higher than PRLr or PRL. These NSC markers initially showed high expression, which gradually declines in tandem with advancing neurodevelopment (Supplementary Figure S1A). By analyzing these markers, we established a temporal framework for the emergence of neural population, enabling a comparison of PRLr expression with key stages of neural differentiation. Conversely, the immature pan-neural marker β-tubulin III progressively increased, ultimately displaying expression levels at least 900 times higher than PRLr (Supplementary Figure S1B). Similarly, the mature pan-neural marker Map2 exhibited a steady increase, becoming the second most highly expressed gene among those evaluated.

Eomes (also known as Tbr2) and Tbr1 were selected as representative markers of intermediate progenitors and early deep-layer neurons, respectively, because they are part of a well-characterized transcriptional hierarchy regulating cortical excitatory neurogenesis. This hierarchy proceeds in an order of progression from Emx1 to Eomes, Tbr1 and ultimately Satb2 between E11 and E18 (La Manno et al., 2021). Eomes and Tbr1 remained stable from E10.5 to E16.5, while the mature pan-neural marker NeuN and the cortical layer markers Ctip2 (deep layers) and Satb2 (upper layers) gradually rose, reflecting expected temporal differences during corticogenesis (Supplementary Figure S1A). Gfap, a pan-glial cell marker, showed negligible expression until E16.5 (Supplementary Figure S1A), in line with the later onset of gliogenesis (Zhang et al., 2020). Collectively, these profiles highlight the tightly regulated progression of neurogenesis during early development and places PRLr expression dynamics in context, suggesting a potential role for PRLr in neural differentiation and cortical organization.

Because epigenetic modifications greatly influence gene expression in neural development, we explored the chromatin landscape of the PRL and PRLr loci using published ATAC-seq and ChIP-seq data from mouse fetal development (Gorkin et al., 2020) (Supplementary Figures S1C–F). At the PRLr locus, the active enhancer marker H3K27ac and the silencing marker H3K27me3 showed dynamic changes from E10.5 to E16.5. H3K27ac was low at E10.5, rose at E11.5, decreased between E12.5 and E13.5 and increased again at E14.5, localizing to a single peak at E15.5 (Supplementary Figure S1C). H3K27me3 initially peaked near the gene start at E10.5, spread across the locus at E11.5 and gradually declined afterward (Supplementary Figure S1D). By E16.5, accessibility at the PRLr locus rose again, mirroring E11.5 levels. These patterns imply active regulation of PRLr to sustain low but increasing expression, although complete removal of H3K27me3 did not appear necessary for PRLr upregulation.

ATAC-seq analysis indicated low chromatin accessibility at the PRL locus, with a stable peak from E13.5 to E16.5 (Supplementary Figure S1E). Prl3b1, the most highly expressed PRLr ligand, demonstrated decreasing accessibility commensurate with its declining expression from E10.5 to E12.5. Similar patterns emerged for other placental lactogens (Prl3d1, Prl3d2, Prl3d3, Prlc1, Prl3b1, Prl3a1). In contrast, the PRLr locus displayed three stable accessibility peaks between E13.5 to E16.5, alongside an additional peak at E11.5 near the transcription start site and another peak by E12.5, which correlated with its rising expression. These observations highlight the nuanced epigenetic orchestration involved in PRL and PRLr during critical neurodevelopmental stage.

To confirm PRLr presence, we performed immunofluorescence on E12.5 mouse embryos, a stage characterized by NSCs and early immature neurons in the FB (Supplementary Figure S2). PRLr staining was evident in the FB, midbrain, and hindbrain, including strong signals in the telencephalic vesicle, the precursor to the cerebral cortex, notably within the superficial layer of the dorsal pallium. PRLr appeared more pronounced in the midbrain and hindbrain than in the FB and colocalized with Nestin and β-tubulin III in all regions, reinforcing its presence in both NSCs and differentiating neurons essential for cortical neurogenesis.

In summary, despite low PRL expression during these developmental stages, likely reflecting repressed chromatin, the dynamic regulation of PRLr suggests it is nevertheless important in early neurogenesis and FB development, especially given the marked rise in PRLr at later stages. The minimal expression of PRL and other PRL-like proteins implies that extrinsic ligands, possibly maternal in origin, may fine-tune developmental processes through PRLr engagement.

### Dynamic expression of prolactin receptor during cortical neural differentiation of mouse embryonic stem cells to cortical neurons

Given the identification of PRLr in the FB during mouse embryo development, we sought to examine PRLr expression during an *in vitro* cortical neuron differentiation protocol derived from mESC (Gaspard et al., 2009; Gaspard et al., 2008).

To confirm the undifferentiated state of the mESCs, we initially observed the characteristic dome-shaped colonies with defined borders and compact cellular structure. These colonies stained positively for Oct4, Sox2, and Nanog, confirming their pluripotent state (Supplementary Figures S3A, B). During differentiation process, we observed expected morphological transitions, including a shift from star-shaped cells forming irregular colonies (days 1–6) to neural rosettes and cells with neurites (days 14–21), indicating neural onset and maturation (Supplementary Figure S3C).

We screened the PRLr expression through daily sampling during differentiation (Figures 1B, C). PRLr expression was highest at D0 in undifferentiated cells, followed by a pronounced decrease by Day 1. This reduction persisted until Day 5, coinciding with the exit from pluripotency, a phase marked by metabolic and cellular remodeling that underpins cell fate decisions (De Belly et al., 2021; Mathieu and Ruohola-Baker, 2017). This low expression persisted until day 15, with a brief increase between days 6–12, corresponding to the neurogenic peak and the rise of NSCs (Gaspard et al., 2008). Expression returned to levels comparable to the undifferentiated state by day 18 (Figure 1C). For this analysis, primers specific to the long isoform of the PRLr gene were used, excluding the three short isoforms present in mice.

Next, we used immunofluorescence to track PRLr throughout differentiation from mESCs (Oct4+) to NSCs (Nestin+) to immature neurons (β-tubulin III+) and mature neurons (NeuN+) (Figures 1D–G; Supplementary Figure S4). On day 0, nearly all Oct4+ cells co-expressed PRLr, with signals for both markers decreasing gradually until day 7 (Figure 1D; Supplementary Figure S4A). In the NSC, PRLr was detected in Nestin-positive rosettes, although overall PRLr levels remained low from days 6–12, with significant co-localization with Nestin. A spatial separation between PRLr and Nestin emerged by days 13 and 14 (Figure 1E; Supplementary Figure S4B). Interestingly, PRLr and β-tubulin III were primarily co-localized in cell neurites, with low PRLr expression in immature neurons from days 13–18 (Figure 1F; Supplementary Figure S4C). In mature neurons, two NeuN + populations were identified: one with low PRLr signal and another with high PRLr levels, particularly in the neurites (Figure 1G; Supplementary Figure S4D).

To quantify PRLr co-localization with these markers, we analyzed immunofluorescence intensities using confocal micrographs and generated overlay plots for each protein (Figure 1H) (Weberling and Zernicka-Goetz, 2021). Co-localization between Oct4 and PRLr at day 0 confirmed their presence in undifferentiated mESCs, with a concurrent decline in both markers as differentiation progressed until day 7. For Nestin (analyzed from days 6–14), we observed peak intensity at day 7, with sustained high levels through day 12 and a notable divergence from PRLr by day 14. For β-tubulin III and NeuN signals increased from days 14–17, though PRLr levels remained consistently lower than those of neural differentiation markers from day 17 onwards (Figure 1H).

Overall, our data suggest that PRLr expression is dynamically regulated during the differentiation of mESCs into cortical neurons. PRLr appears to support pluripotency and NSCs self-renewal, with its function evolving as neural cells differentiate. The observed shifts in PRLr distribution likely indicate its varying roles across cell types during cortical differentiation. Notably, the presence of PRLr in β-tubulin III and NeuN-positive cells, particularly in neurites, suggest a potential additional role in neurite formation and neural maturation.

### Prolactin promotes neurogenesis and neuronal maturation during mESC differentiation

Upon observing the dynamic presence of PRLr during the differentiation of cortical neurons we sought to determine the effects of PRL administration during early differentiation (Ed) or late differentiation (Ld) phases following our protocol. To this end, we validated the differentiation protocol by analyzing markers at specific days of *in vitro* differentiation (DIV): Sox2 (16.88% at day 12), Nestin (30.42% at day 12% and 55.73% at day 14), β-tubulin III (2.17% at day 12), Tbr1 (3.12% at day 21), NeuN (14.22% at day 21% and 41.39% at day 28), Map2 (28.45% at day 21% and 25.79% at day 28), and Gfap (2.2% at day 21 and 12.17 at day 28) (Supplementary Table S1). These values corroborated the efficiency of our differentiation protocol, however with light differences in some phenotypes analyzed in comparison with previous reports (Gaspard et al., 2008; Sadegh and Macklis, 2014).

Next, we administered PRL at various concentrations (0.2, 0.4, 2, 6 and 20 nM) during Ed or Ld to evaluate the effect on neuronal markers at day 12 and 21. During early differentiation, PRL treatment showed a trend toward an increased percentage of Nestin and Sox2-positive cells at PRL concentration ranging from 2 nM to 20 nM; however, these results were not statistically significant compared to the control group (Supplementary Figure S5).

**FIGURE 2:**
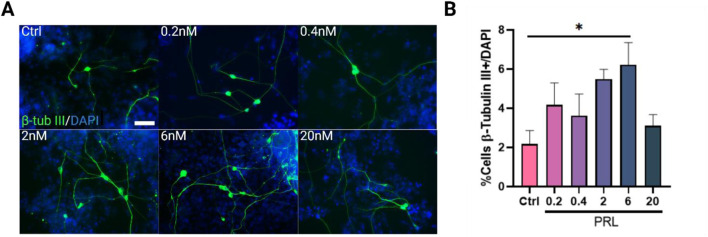
Prolactin increases β-tubulin III-positive cells in early differentiation, primarily at 6 nM. **(A)** Representative images of β-tubulin III (green)/DAPI (blue) positive cells in control and PRL-treated groups, across concentrations from 0.2 to 20 nM. Images were acquired using identical gain and exposure settings on an epifluorescence microscope. Scale bar: 25 μm. **(B)** Quantification of β-Tubullin III-positive (immature neurons) cells at day 12 of differentiation. PRL treatment increased β-Tubullin III-positive cells at all concentrations, with 6 nM yielding significant statistical difference. Bar plot, mean ± SEM. Tukey´s multiple comparisons test, *p* < 0.05. F, DFn, Dfd values: 2.854, 5 and 18. Data represent three replicates analyzed in duplicate.

The lack of an observed effect on NSCs, despite their PRLr positivity and high correlation in signal intensities, led us to investigate whether PRL influences the progeny of NSCs. Interestingly, when cells were treated with 6 nM PRL during the Ed phase, there was a significant increase in the percentage of β-tubulin III-positive immature neurons compared to the control, with PRL-treated cultures exhibiting at least three times more neurons than controls (Figures 2A, B). Given this effect on β-tubulin III-positive cells at 6nM, we further assessed PRL’s influence on NSC proliferation using an EdU assay at day 14 (Supplementary Figure S6). Following a 1-h EdU incubation, no significant differences were found in EdU-positive cell counts, Nestin optical density, EdU and Nestin co-localization, or total Nestin-positive cell counts (Supplementary Figures S6C, F). Moreover, PRL did not affect NSC proliferation rates between days 12 (30.42% Nestin + cells) and 14 (55.73% Nestin + cells), where a doubling of NSC numbers was observed as expected in the control (Supplementary Table S1; Supplementary Figures S5B, S6F).These findings suggest that while PRL may not significantly impact NSC maintenance, it promotes immature cell differentiation without reducing the NSC pool, possibly affecting other cell types that were not evaluated in this study. This inference is further supported by the absence of differences in total cell numbers at day 12 between control and 6 nM PRL-treated groups (Supplementary Figure S7A).

**FIGURE 3:**
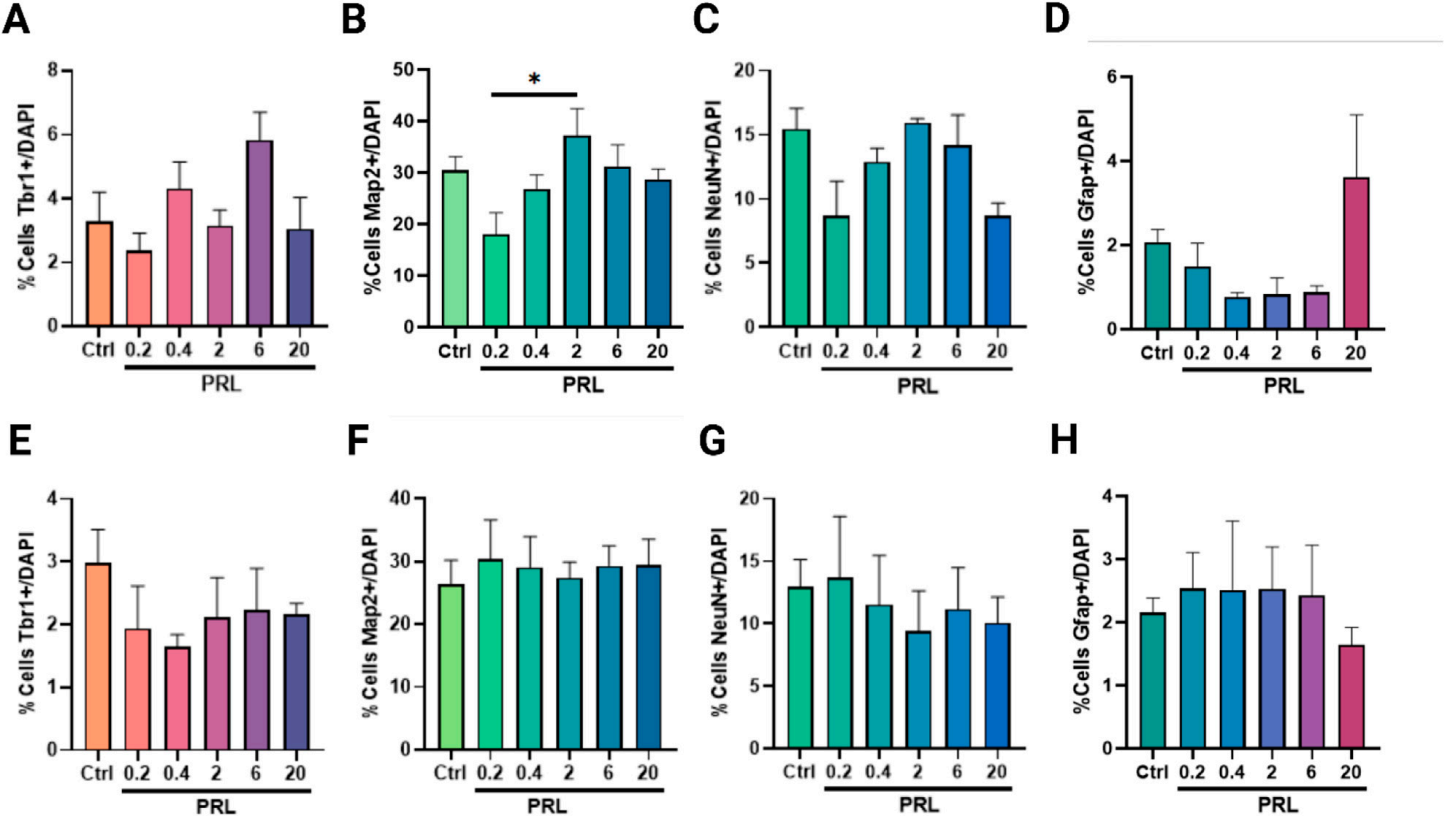
Temporal specificity of PRL in increasing neuronal populations. Quantification of neuronal cells classes at day 21 following PRL treatments during early differentiation (day 0–12) **(A–D)** and late differentiation (day 13–21) **(E–H)**. **(A)** Tbr1-positive cells showed a slight increase trend with 6nM, but PRL did not reach significance. **(B)** Map2-positive cells increased between 0.2 and 2 nM PRL. Bar plot, mean ± SEM. Tukey’s multiple comparisons test, *p* = 0.0385. F, DFn, Dfd values: 2.999, 5 and 18. **(C)** NeuN-positive cells remained unaffected by PRL at all concentrations. **(D)** No effect was observed in GFAP-positive cells. **(E–H)** No significant PRL effect during late differentiation on **(E)** Tbr1, **(F)** Map2, **(G)** NeuN or **(H)** Gfap.

We further evaluated the effect of PRL on Tbr1 (deep-layer cortical neurons), Map2, NeuN, and Gfap during both Ed and Ld at day 21. During Ed, PRL treatment showed a fluctuation, with Tbr1+ cells percentages similar to the control, but a trend toward an increase was observed only at 6 nM (Figure 3A). A bell-shaped dose-response pattern was observed for mature neuron markers NeuN and Map2, peaking around 6 nM and decreasing at higher (20 nM) and lower (0.2 nM) concentrations (Figures 3B, C). This decrease created a statistically significant difference between 0.2 and 6 nM of PRL on Map2+ cells (Figure 3B). Additionally, the total number of DAPI + cells at day 21, did not differ across all the conditions evaluated (Supplementary Figure S7B), suggesting that the observed effect was not due to cell death or proliferation.

The bell-shaped dose pattern observed in mature neurons with the administration of PRL during early differentiation was inverted when Gfap + cells were evaluated (Figure 3D). However, no statistically significant differences were found for any concentrations or markers tested compared to the control (Figures 3A–D). Similarly, PRL treatment during late differentiation did not induce significant changes in marker number across all concentrations tested compared to the control (Figures 3E–H).

**FIGURE 4:**
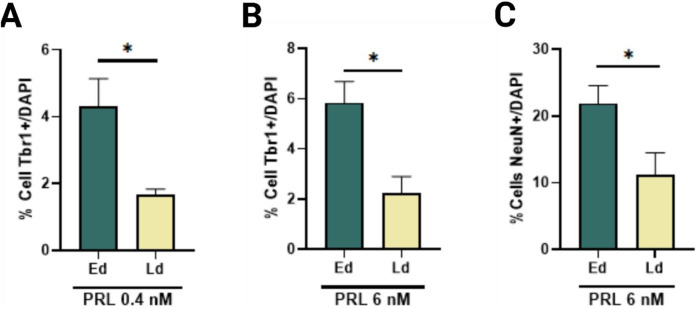
PRL enhances the Tbr1 and NeuN-positive cells in a time-specific manner. **(A)** Comparison of Tbr1-positive cells at day 21 post 0.4 nM PRL treatment, showed increased Tbr1 increased number in early differentiation versus late differentiation, Bar plot, mean ± SEM. Unpaired t-test, *p* < 0.05, *p* = 0.0195. F, DFn, Dfd values: 20.4, 3 and 3. **(B)** Tbr1-positive cells increased following 6 nM PRL during early differentiation but not late differentiation. Bar plot, mean ± SEM. Unpaired t-test, *p* < 0.05, *p* = 0.0155. F, DFn, Dfd values: 1.677, 3 and 3. **(C)** NeuN-positive cells also increased significantly with 6 nM PRL in early but not late differentiation. Bar plot, mean ± SEM. Unpaired t-test, *p* < 0.05, *p* = 0.0452. F, DFn, Dfd values were: 1.536, 3 and 3. Data from three replicates analyzed in duplicate.

Given the lack of statistically significant changes between PRL concentrations in Ed and Ld, only patterns and tendences observed with treatment on Ed and considering the complex regulation observed in the receptor during the protocol, we compared the effects of PRL across the same phenotypes but with Ed and Ld as variables. We observed a significant increase in Tbr1+ cells at 0.4 nM and 6 nM during Ed (Figures 4A, B), as well as a significant increase in NeuN + cells as 6 nM (Figure 4C). These findings suggest that PRL has a stage-specific effect on neuronal differentiation, particularly enhancing Tbr1 and NeuN expression during early differentiation.

### Prolactin enhances dendritic complexity in cultured neurons derived from mESC

Following the observed increase in β-tubulin III, NeuN and Tbr1-positive cells with 6 nM PRL treatment during Ed, we selected this concentration to mimic physiological levels relevant to neurogenesis and neuritogenesis in later neurodevelopmental stages (Martínez-Alarcón et al., 2022; Pathipati et al., 2011). To clarify PRL’s physiological role in corticogenesis and its enrichment in neurites, we investigated whether PRL enhances dendritic complexity.

**FIGURE 5:**
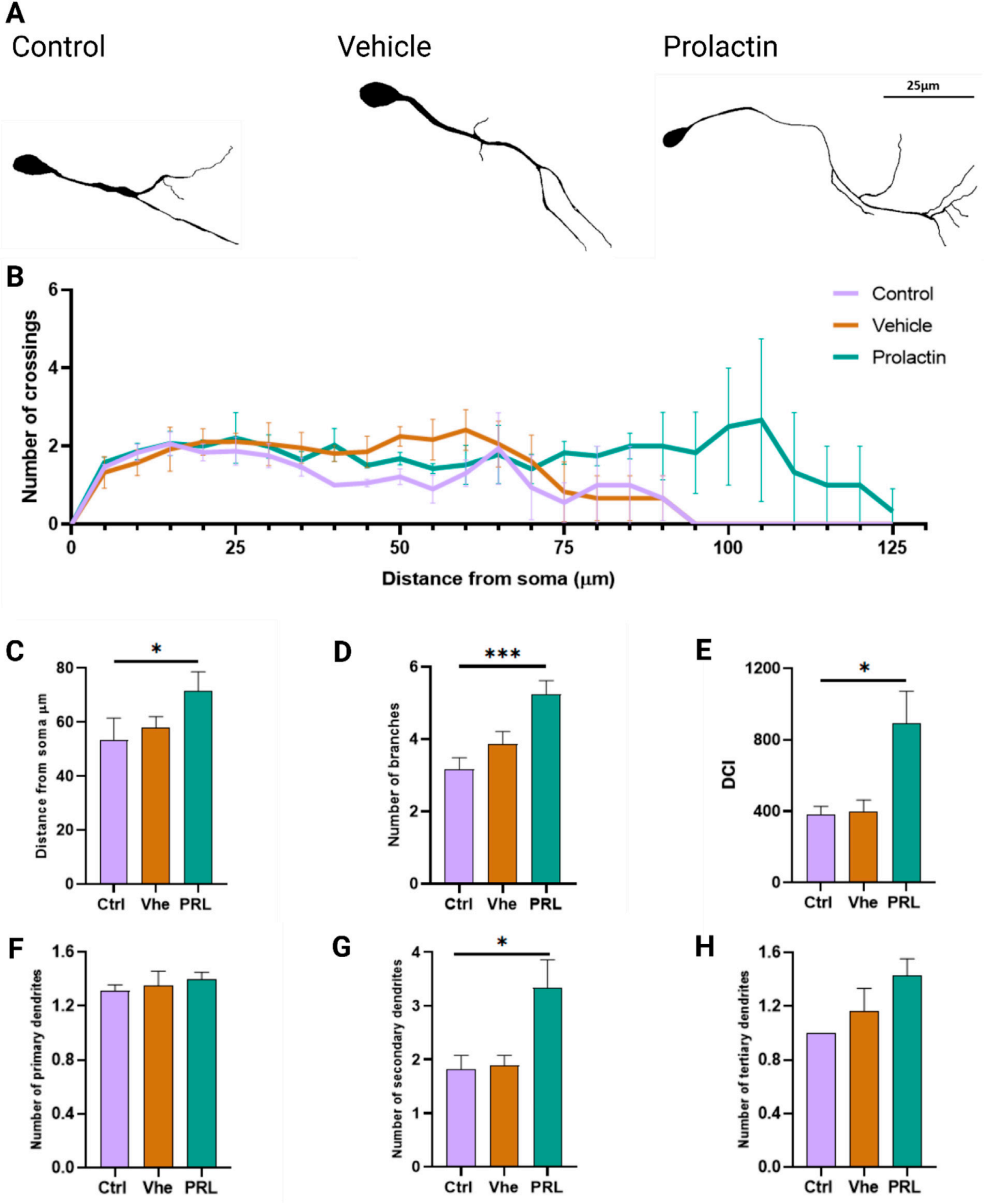
Prolactin increases dendritic length and complexity in cortical neurons. **(A)** Representative tracings from phase-contrast micrographs of neurons at day 14 of differentiation. Scale bar: 25 μm. **(B)** Sholl analysis showing dendritic intersections from the soma in three groups. n = 32 neurons per group from 3 biological replicates. **(C)** Distance from soma analyzed showed increased length in the 6 nM of PRL group. Bar plot, mean ± SEM. Tukey’s multiple comparison test, *p* = 0.0373. F, DFn, Dfd values: 5.979, 2 and 6. **(D)** PRL at 6 nM also increased branching points Tukey’s multiple comparison test, *p* = 0.0001. F, DFn, Dfd values: 1.306, 2 and 100. **(E)** Dendritic complexity index (DCI) analysis showed enhancement with 6 nM PRL. Tukey’s multiple comparisons test, *p* = 0.0312. F, DFn, Dfd values: 6.534, 2 and 6. **(F)** Quantification of primary dendrites showed no significant differences. **(G)** Secondary dendrites were also increased by 6 nM PRL. Tukey’s multiple comparisons test, *p* = 0.0373. F, DFn, Dfd values were: 5.980, 2 and 6. **(H)** Analysis of tertiary showed no significant PRL effect.

In this neuritogenesis assay, we evaluated primary dendrite length, number of crossings and dendritic complexity through Sholl’s analysis (Sholl, 1953). PRL-treated neurons exhibited greater morphological complexity than control group (Figure 5A). Specifically, PRL treatment led to significant longer primary dendrites, with a maximum observed difference of 30 μm between the PRL and control groups (Figures 5B, C), resulting in a 0.8-fold increase in the area under the curve (Supplementary Figure S8A). PRL also increase branch points by 1.6-fold, further demonstrating its impact on dendritic complexity (Figure 5D). No differences in the number of crossings were observed (Supplementary Figure S8B). PRL treatment notably increased the proportions of neurons with dendrite lengths between 80 and 125 µm (bin 3) and decreased those with shorter dendrites (<40 μm, bin 1), while no changes were seen in the intermediate group (40–80 μm, bin 2) (Supplementary Figure S8C).

These results suggest that PRL-treated neurons may form more extensive connections, as evidenced by an increase in the dendritic complexity index (DCI), which measures a neuron’s capacity for synaptic connectivity. PRL-treated neurons showed a 2.32-fold higher DCI compared to controls (DCI, Ctrl: 383.28 vs. PRL: 891.67) (Figure 5E). While PRL did not affect the number of primary and tertiary dendrites (Figures 5F, H), it significantly increased the number of secondary dendrites by 1.84-fold compared to controls (Figure 5G).

These findings suggest that PRL enhances dendritic complexity and length when applied during the peak of neuronal differentiation surge in this protocol, primarily by increasing secondary dendrites numbers. This effect implies that PRL not only supports neuronal differentiation but also augments dendritic complexity, potentially facilitating neural maturation and functional integration in early differentiation. Thus, PRL may act as a factor that enhances structural and functional neural plasticity, impacting neuronal signaling efficiency and specificity during corticogenesis.

### Prolactin maintains GFAP + cell populations while modulating protoplasmic astrocyte derivation

Since the percentage of GFAP + cells at day 21 showed no significant differences between the control and PRL-treated groups (Figures 3D, H), we hypothesized that the protocol’s late onset of gliogenesis may have limited the detection of PRL’s effects on astrocytes. To further explore PRL’s influence on astrocyte populations, we extended the analysis to day 28, when gliogenesis become more pronounced, with an increase in GFAP + cells from 2.2% at day 21% to 12.17% at day 28 (Supplementary Table S1).

**FIGURE 6:**
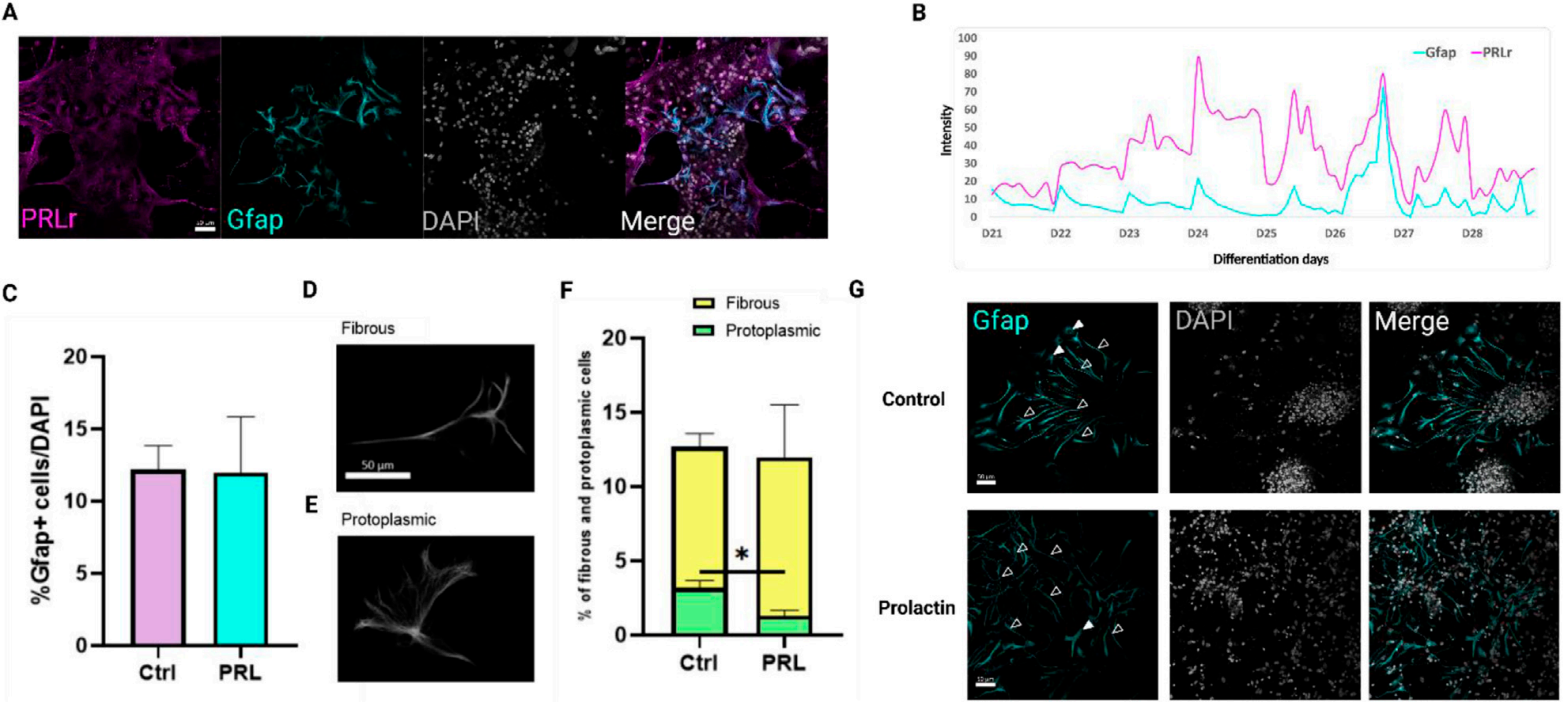
Prolactin modulates astrocyte morphology without affecting total Gfap + cells. **(A)** Confocal image showing PRLr (magenta) localization in GFAP+ (cyan) astrocytes at day 28 of differentiation. Scale bar: 50 μm. **(B)** Merged plot of PRLr and GFAP profiles from day 21 to day 28, highlighting temporal expression in neural differentiation. Intensity analyzed with Fiji and Zen Lite software. **(C)** Percentage of GFAP + cells relative to DAPI + cells show no significant differences between control and PRL-treated groups. **(D, E)** Representative images of fibrous and protoplasmic astrocytes. **(F)** Morphology analysis confirmed decreased protoplasmic astrocytes proportions with PRL treatment, without affecting fibrous astrocytes values are mean ± SEM; Tukey’s test (*p < 0.05; *p* = 0.0354). F, DFn, Dfd values: 1.758, 2, and 2. **(G)** Immunofluorescence images of GFAP+ (cyan) astrocytes in both control and PRL-treated samples, highlighting distinct morphological differences. Fibrous astrocytes are marked with open arrowheads, while protoplasmic astrocytes are indicated with filled arrowheads. Scale bar: 50 μm.

To investigate whether GFAP + cells co-localize with PRLr, we identified double-positive cells at day 28, with both markers predominantly localized in the cytoplasmic membrane (Figure 6A). Temporal immunostaining revealed PRLr co-localization with GFAP in astrocytes, with PRLr signals observed in cytoplasmic extensions at lower intensities. From day 21 to day 28, PRLr co-localized with GFAP + cells, displaying a primarily cytoplasmic distribution pattern (Figure 6B; Supplementary Figure S4E). PRLr intensity peaked between days 24 and 28, while GFAP remained low with a slight peak on day 26 and an increase in positive cell number from day 21–28 (Figure 6B; Supplementary Figure S4E).

This extended timeframe allowed for a more detailed analysis of astrocyte maturation and morphological changes. By day 28, the GFAP + cells percentage remained unchanged between control and PRL-treated groups, indicating PRL does not significantly affect astrocytes numbers (Figure 6C). However, a significant observation arose when examining astrocytes morphology, particularly protoplasmic versus fibrous subtypes (Figures 6D, E). Protoplasmic astrocytes, prevalent in cortical gray matter and known for their highly branched processes critical to synaptic modulation and blood flow regulation (Tabata, 2015), showed a marked reduction in PRL-treated cultures compared to controls (Figures 6F, G). This suggests PRL may selectively influence astrocyte maturation or activation shifting glial function away from synaptic interaction and blood flow modulation.

Conversely, fibrous astrocytes (Figure 6D), typically found in white matter and associated with blood-brain-barrier maintenance and ionic homeostasis (Tabata, 2015), did not show significant differences between groups (Figures 6F, G). As fibrous astrocytes are generally considered less mature or active than protoplasmic astrocytes, these findings suggest PRL may delay astrocyte maturation, aligning with the observe increase in neuronal markers (Figures 2, 4).

These results support our earlier findings that PRL promotes neurogenesis, particularly in early differentiation phases, potentially at the expense of gliogenesis. The reduction in mature protoplasmic astrocytes, with an unchanged fibrous astrocytes population, further suggest PRL may shift the balance between neuronal and glial differentiation, during cortical development stages.

In summary, while PRL does not significantly impact the total number of GFAP + cells, it appear to influence astrocyte subtype distribution and maturation, potentially delaying protoplasmic astrocyte maturation. This mechanism may allow PRL to enhance neurogenesis and early neural circuit formation by modulating glial cell maturation during corticogenesis.

### Prolactin signaling and its association with neuroactive ligand-receptor interactions

Given the low yet active expression of PRLr alongside several ligands that interact with it, we explored potential pathways related to neurodevelopment in the FB using datasets from Peng He. By comparing data from E10.5 to 13.5 and E10.5 to16.5, our analysis revealed an enrichment of two gene sets, each comprising 307 genes involved in the neuroactive ligand-receptor interaction pathway. Notably, PRLr appeared in both temporal comparisons, along with genes associated with GH signalling, including the GH receptor (Supplementary Figure S9A). The presence of GH signalling genes aligns with the known role of GH in neural growth, differentiation, neuroprotection and synaptogenesis (Bianchi et al., 2017; Martínez-Moreno and Arámburo, 2020).

Moreover, our ATAC-seq and ChiP-seq analyses consistently indicated an enrichment of PRL when comparing E16.5 to E10.5 (Supplementary Figure S9A), suggesting that PRL may act as an endogenous ligand for PRLr during these critical stages of neurodevelopment. It is also possible that maternal PRL or PRL from other embryonic tissues contributes to this interaction (Supplementary Figures S9B, C). These findings support the hypothesis that PRL and its receptor play an essential role in FB and cortical development which highlighting the complexity of PRL signaling during neurodevelopment.

## Discussion

While PRL has been extensively studied in adult animals, its potential influence on critical events such as pluripotency and corticogenesis during embryonic development remains poorly understood. This is particularly significant given the elevated levels of PRL in the bloodstream and widespread distribution of its receptor at these stages. In this study, we present evidence for PRL’s involvement in embryonic neurogenesis and the maturation of neuronal and glial populations derived from mESCs via a neural differentiation protocol.

PRLr is a multifaceted receptor capable of interacting with diverse ligands, modulating numerous cellular processes through distinct signaling pathways (Brooks, 2012). In early development, PRLr expression begins at the oocyte stage, continues through the blastocyst phase (Kiapekou et al., 2005) and is detected in NSC, which are exposed to high concentrations of PRL, sourced from the decidua and amniotic fluid (Rosenberg et al., 1980), This distribution suggests that PRL and its receptor may play key regulatory roles in embryonic developmental process.

In investigating PRL and PRLr roles in FB development from E10.5 to 16.5, several findings emerged. Transcriptome analysis of mouse embryo dataset (He et al., 2020) demonstrated PRLr expression in FB during this embryonic period with a notable increase in expression at E16.5 compared to E10.5 (Supplementary Figure S1A). NSC markers like Sox2, Nestin and Pax6 were also highly expressed, although Eomes and Tbr1 levels remained stable throughout. β-tubulin III, a neuronal marker, showed the highest expression levels, progressively increased. PRL expression itself remained consistently low during this period (Supplementary Figure S1B), as did PRL-like proteins capable of binding to PRLr (Supplementary Figure S1C).

Despite fluctuations in PRLr expression, the PRL gene’s epigenetic landscape remained relatively stable. ATAC-seq and Chip-seq analyses (Gorkin et al., 2020) revealed low levels of histone marks, such as H3K27ac and H3K27me3, at both the PRL and PRLr loci in the FB, suggesting a chromatin environment progressively conducive to expression (Supplementary Figure S1D).This persistence of epigenetic marks, despite changes in PRLr expression, implies a complex regulatory mechanism in modulating gene expression without substantial chromatin state alterations, highlighting the interaction between transcriptional activity and epigenetic modifications during neural development. Analyzing additional histone modifications, including H3K4me1, H3K9ac, H3K36me3, H3K9me3, H3K27ac and H3K27me3, would further support the existence of a finely tuned regulatory environment that maintains gene accessibility for activation during development.

The low levels of these modifications at the PRLr gene suggest a transcriptionally permissive but not highly active state, indicating that while PRLr is poised for expression, it may not be heavily regulated by these epigenetic marks during critical neurodevelopmental stages. These findings suggest dynamic regulation of PRLr expression during FB development, with a significant increase at later stages, likely reflecting the receptor’s role in responding to developmental cues essential for neurogenesis. Additionally, the consistently low expression of PRL and related PRL-like proteins suggest that PRL and potentially other extrinsic ligands, such as cytokines or growth factors, might interact with PRLr. The detection of PRLr during early murine brain development, particularly in the FB at E12, suggests that such interactions may subtly but crucially influence developmental process by providing essential signals for neural maturation and differentiation. Understanding these interactions may reveal the regulatory networks that ensure proper neural development.

To explore the hypothesis that PRLr is a critical spatiotemporal regulator during early embryonic development, particularly during neurogenesis, we performed a series of experiments using a cortical differentiation protocol in mESC. Initial results demonstrated PRLr co-expression with pluripotency and NSC markers, suggesting a dual role in regulating pluripotency and early neurogenesis (Supplementary Figures S4A, B). PRLr was highly expressed in undifferentiated mESC, co-localizing with Oct4 at day zero, indicating PRL’s possible role in maintain pluripotency. As differentiation progressed, both Oct4 and PRLr expression decreased, indicating a transition from a pluripotent to a more differentiated state, where PRLr and its ligands may be essentials, reflecting their role in diapause *in vivo*.

We observed that PRLr expression decreased during *in vitro* differentiation, supporting its role in the transition from pluripotent to neural cells. This finding aligns with the sustained PRLr expression observed in the FB from E10.5 to E16.5, where it co-expressed with markers of neural progenitors and developing neurons (Supplementary Figures S1, S2). Variations in PRLr expression has been documented in other tissues, including the uterine epithelium and corpus luteum, where they respond to molecular changes in the extracellular environment (Eyal et al., 2007; Fenelon et al., 2014). In the mammary gland epithelium, PRLr expression also fluctuate in response to hormonal shifts during pregnancy and lactation (Meng et al., 2023; Varas and Jahn, 2005), highlighting its sensitivity to extracellular signals cues. Similarly, during diapause, PRLr is highly expressed in the corpus luteum, decreasing during embryo reactivation and increasing upon implantation (Fenelon et al., 2014). In mammary gland epithelium, PRLr expression declines during high progesterone levels in pregnancy and increases *postpartum* to promote lactogenesis (Atıcı et al., 2021; Goldhar et al., 2011; Nishikawa et al., 1994). These examples demonstrate that PRLr expression respond to significant molecular changes in the extracellular environment, affecting ligand availability for receptor regulation.

Notably, the peak in Nestin expression at day 7, with continued PRLr co-localization, highlights PRLr’s involvement in the early stages of neural *in vitro* differentiation, specifically within the NSC population (Supplementary Figure S4). Co-localization persisted through day 12, suggesting PRLr’s role in NSC maintenance and proliferation. When examining PRL’s potential effects on NSC, we found that NSCs co-expressed PRLr; although, cell number and proliferation remained unaffected by PRL treatments. The presence of PRLr in Nestin-positive cells has been reported in adults, where it play a role in regulating neurogenesis under conditions such as pregnancy (Shingo et al., 2003) and chronic stress (Torner, 2016).

A key point from this study is the discovery of two NeuN + neuron population distinguished by low versus high PRLr expression. This distinction likely signifies different maturation or functional states, suggesting that PRLr might play specific roles in regulating the maturation and integration of excitatory neurons.

PRL expression did not align with later-stage neural markers, such as β-tubulin III and NeuN. These markers increased at days 14 and 17, respectively, while PRLr intensity decreased (Supplementary Figure S4), suggesting that PRLr may not be essential for later neural maturation stages or that its role shift as neural cells differentiate (Figure 1H; Supplementary Figure S4). These observations highlight the dynamic nature of PRLr expression during neural development and underscore the role of epigenetic stability in ensuring precise regulation during differentiation and maturation.

Given that no significant differences in NSC proliferation were observed from days 12–14, we suggest that PRL does not affect the cell cycle or of G1 phase length (Liu et al., 2019). Interestingly, PRL significantly increased the number of β-tubulin III + cells, suggesting a role in early neurogenesis with effects dependent on concentration and timing. Previous studies have shown varying effects of PRL on neuronal populations; in human primary neuron cultures, PRL increased neurite quantity (Tani et al., 2024), through no changes in dendritic maturation were observed in hippocampal neurons (Smeeth et al., 2021). PRL has also been linked to regulating genes involved in neuronal development and synaptic function (Cabrera-Reyes et al., 2019). These findings suggest that PRL’s potential role in enhancing neuronal survival and maturation through mechanisms distinct from cell proliferation.

The early differentiation of Tbr1+ neurons appears to be primarily governed by developmental timing rather than a Tbr1-specific PRL signal pathway. This suggest that PRL may influence the broader temporal regulation of neural maturation rather than acting through a lineage-specific mechanism. PRL’s role in orchestrating the orderly progression of cortical neuron development is particularly intriguing, as its effects may be mediated through interactions with other neurogenic signals or epigenetic modifications tant regulate the differentiation. However, the precise mechanism underlying this regulation remains to be elucidated.

Astrogliogenesis is crucial for cortical development as astrocytes support and regulate neural function. During this process, astrocytes aid in synapse formation, which is essential for proper neural circuitry. Our analysis of the glial population revealed that PRL influence astrogliogenesis and glial morphology, particularly by reducing the protoplasmic phenotype compared to controls (Figure 6). PRL’s interactions with astrocytes, particularly regarding brain injury, inflammation and neuropathological processes, are well-documented (Anagnostou et al., 2018; Castillo et al., 2024; Ramos-Martinez et al., 2021).

Most studies on PRL’s role in astrogliogenesis focus on adult or juvenile organisms, making our findings particularly relevant for earlier development. PRL may also influence NSC before astrogliogenesis, potentially altering their gliogenic processes. By day 21, gliogenesis percentages were low compared to other cell types (Supplementary Table S1) likely due to the delayed transition from NSCs to astroglia, occurs around embryonic day 16 in murine embryos (Clavreul et al., 2022). Consequently, PRL’s effects became more apparent at later stages of the protocol (day 28) (Figure 6).

Understanding PRL’s role in neural development has significant clinical potential. PRL has been associated with neurodevelopmental conditions, making it a target for therapeutic interventions. Low PRL levels correlate with anencephaly (Winters et al., 1975), while high maternal serum PRL levels are linked to growth retardation and anencephaly (Arosio et al., 1995). Furthermore, PRL’s link to maternal diabetes, associated with neurodevelopmental defects, emphasizes its relevance (Ekinci et al., 2017; Li et al., 2020; Rassie et al., 2022). Recently, reduced PRL/PRL-like protein levels were associated with small-for-gestational-age fetal growth in mice (Lopez-Tello and Sferruzzi-Perri, 2023). Clarifying the molecular mechanisms by which PRL influences NSC at the genetic and epigenetic levels, by promoting neurogenesis, enhancing morphological maturity, delaying gliogenesis, or impacting glial maturation—may inform therapies aimed at supporting neurodevelopment.

A significant limitation of this study is the lack of specificity regarding PRLr isoforms in cells and the signalling pathways involved. While our *in vitro* model is informative, *in vivo* validation would offer more comprehensive insights. Given PRLr promiscuity with growth hormone and PRL-like proteins, these interactions may contribute to the observed effects. Other regulatory mechanisms, distinct from cell proliferation, could contribute to the observed increase in specific neuronal populations and enhanced morphological maturity. Although direct evidence linking PRL to mechanisms such as the epigenetic predisposition of NSCs to neuronal differentiation, or an increased expression of genes involved in cytoskeleton remodelling, neurite growth, or other pathways beyond cell proliferation is still limited, these factors way explain our observations.

In summary, our findings underscore PRL and PRLr’s crucial role in pluripotency, early neurogenesis, and neuronal maturation. The findings also lay groundwork for future studies on epigenetic and molecular regulatory pathways, extending understanding of PRL’s broader biological roles across different systems. These results collectively underscore PRL’s potential as a significant factor in neurodevelopment and a promising target for clinical applications.

## Data Availability

The datasets presented in this article are not readily available because NA. Requests to access the datasets should be directed to Nestor Fabian Diaz nfdiaz00@yahoo.com.mx.

## Supplementary material

SUPPLEMENTARY FIGURE 1 Bioinformatic analysis of transcriptional and epigenetic landscape of PRL and PRLr during mouse forebrain development. **(A)** Comparative analysis of gene expression for PRLr, PRL and key neural markers (Sox2, Nestin, Pax6, Eomes, Tbr1, Map2, NeuN, Ctip2, Satb2, and GFAP) from embryonic days E10.5 to E16.5. **(B)** β-tubulin III expression profile, highlighting its dynamic changes during FB development. Histone modification patterns: **(C)** H3K27ac (active enhancers) and **(D)** H3K27me3 (gene silencing) at the PRLr locus across the embryonic stages analyzed, showing temporal chromatin activity fluctuations. **(E, F)** Chromatin accessibility profiles (ATAC-seq) at the PRL and PRLr loci from E10.5 to E16.5. Placental lactogens (e.g., Prl3a1, Prl3b1) in proximity to the PRL locus exhibit a decrease in accessibility, reflecting their expression dynamics.

SUPPLEMENTARY FIGURE 2 Light sheet fluorescence microscopy images of the developing murine brain at embryonic day 12 (E12). **(A)** PRLr expression in the developing brain. **(B)** Nestin staining of neural stem cells. **(C)** β-tubulin III expression marking immature neurons. **(D)** DAPI staining indicating cell nuclei. **(E)** Overlay of PRLr and DAPI signals, showing the localization PRLr relative to cell nuclei. **(F)** Co-localization of PRLr and Nestin in neural stem cells. **(G)** Co-localization of PRLr and β-tubulin III suggesting PRLr presence in early neurons. **(H)** Merged image showing combined signals from PRLr, Nestin, β-tubulin III, and DAPI. Scale bar: 1,000 μm. HB, hindbrain; MB, midbrain and FB, forebrain.

SUPPLEMENTARY FIGURE 3 Establishment and experimental design of the mESC differentiation protocol. **(A)** Classical morphology of mESCs at day 0, characterized by dome-shaped colonies on a feeder layer. **(B)** Representative immunofluorescence images showing pluripotency markers in undifferentiated mESCs, [Oct4 (green), Sox2 (red), and Nanog (red)] confirming their undifferentiated state. **(C)** Morphological changes during differentiation (days 1 to 21), transitioning from irregular colonies (open arrowheads) to rosette formations (filled arrowheads) and neurite extensions (arrows), marking progression toward neural differentiation. Scale bars: 25 μm. **(D)** Experimental timeline detailing PRL and PRLr evaluation stages -early differentiation (Ed, days 0–12), late differentiation (Ld, days 12–21) and gliogenesis (days 21–28)- and indicating assessed markers (Nestin, Sox2, and β-tubulin III for Ed; Tbr1, Map2, NeuN, and Gfap for Ld and gliogenesis. PRL concentrations (0.2 to 20 nM) and PRLR dynamics are also depicted.

SUPPLEMENTARY FIGURE 4 Representative confocal images showing the temporal co-localization of PRLr with various cell-type markers during mESC differentiation into cortical neurons and glia. **(A)** PRLr localization in Oct4-positive cells from Day 0 to Day 7. **(B)** PRLr colocalization with Nestin-positive NSCs, observed predominantly from Days 6 to 9, with signal persistence through Day 14. **(C)** PRLr expression in β-tubulin III-positive immature neurons, primarily localized to neurites from days 13 to 18. **(D)** PRLr co-localization with NeuN-positive mature neurons from Days 17 to 21, highlighting neurite localization. **(E)** PRLr co-localization with GFAP-positive astrocytes from Days 22 to 28, with a dynamic expression pattern predominantly in cell bodies and reduced signal intensity in cytoplasmic extensions. Images were acquired using confocal microscopy. Scale bar: 50 μm.

SUPPLEMENTARY FIGURE 5 Prolactin does not affect NSCs population. **(A)** Representative immunofluorescence images showing Nestin-positive (green) NSCs in control and those treated with increasing PRL concentrations (0.2, 0.4, 2, 6 and 20 nM) from day 1 to 12. **(B)** Quantification of the percentage of Nestin-Positive cells normalized to DAPI. No statistically significant effect of PRL on NSCs percentage was observed. **(C)** Representative immunofluorescence images of Sox2-positive (red) cells treated with the same PRL concentrations with Sox2 serving as an NSC marker on Day 12. **(D)** Quantification of Sox2-positive cells normalized to DAPI shows no significant difference in NSCs proportion with PRL treatment. Scale bar: 25 μm.

SUPPLEMENTARY FIGURE 6 Prolactin does not significantly impact NSC proliferation. **(A, B)** Representative images of EdU (green) and Nestin (red) positive NSCs at day 14, after treatment with either control **(A)** or PRL **(B)**. Scale bar: 25 μm. **(C)** Quantification of EdU+/DAPI+ cells, showing no significant difference between groups. **(D)** Optical density of Nestin-positive cells in arbitrary units (a.u.), indicating no PRL treatment effect. **(E, F)** No significant changes were observed in the percentage of Nestin+/EdU+ cells or total Nestin+ cells. Data are mean ± SEM of three replicates.

SUPPLEMENTARY FIGURE 7 Quantification of total cells during early and late differentiation stages. **(A)** Total number of cells at day 12 of differentiation under control and PRL treatment (6 nM) showing no significant differences. **(B)** Quantification of DAPI-positive cells across early (day 21) and late differentiation stages, with no significant differences observed between groups. **(C)** Total DAPI-positive cell counts at day 28 of differentiation show a significant decrease in PRL-treated samples compared to controls. Data are mean ± SEM from three biological replicates analyzed in duplicate. Statistical analysis was performed using a paired t-test (**p* < 0.05, *p* = 0.0418; t = 2.719, df = 5).

SUPPLEMENTARY FIGURE 8 Prolactin enhances neuronal complexity during early differentiation stages. **(A)** Fold change in the area under the curve (AUC) for crossings with PRL treatment compared to control (Ctrl) and vehicle (Vhe) groups. Statistical analysis revealed significant differences (**p* < 0.05), indicating enhanced neuronal connectivity. **(B)** Quantification of crossings shows a trend towards increased crossings in PRL-treated neurons compared to controls **(C)** Distribution of neurons across dendritic bins: PRL-treated neurons show a higher percentage in bin 3 (>80 μm) and lower in bin 1 (<40 μm), indicating increased dendritic length and complexity. Data are mean ± SEM from three biological replicates analyzed in duplicates.

SUPPLEMENTARY FIGURE 9 Prolactin and its receptor as key regulators of neurodevelopment. **(A)** Gene lists enriched in the neuroactive ligand-receptor interaction pathway during FB development, with genes enriched from E10.5 to E13.5 in the upper table and those from 10.5 to E16.5 in the lower table. Highlighted genes link to PRL and PRLr-mediated signaling. **(B)** KEGG pathway visualization of the neuroactive ligand-receptor interaction pathway focusing on PRL and PRLr interactions, showing changes from E10.5 to E13.5 in the top panel and E10.5 to E16.5 in the bottom panel. Pathview visualization shows PRLr activity, with red shading indicating increased receptor activity in later stages. **(C)** Schematic and triangular plots depict PRL and PRLr expression dynamics from E10.5–E16.5, with PRLr expression (left) progressively increasing in the FB and PRL expression (right) shows corresponding patterns. The adjacent embryonic schematic highlights PRLr signaling within critical brain regions, emphasizing its role in early FB development.
