# Supplementary figures and images for: Prolactin drives cortical neuron maturation and dendritic development during murine embryonic stem cell differentiation
Source: Front Cell Dev Biol. 2025 Feb 26;13:1551090. doi: 10.3389/fcell.2025.1551090 (PMC11897521; doi:10.3389/fcell.2025.1551090)

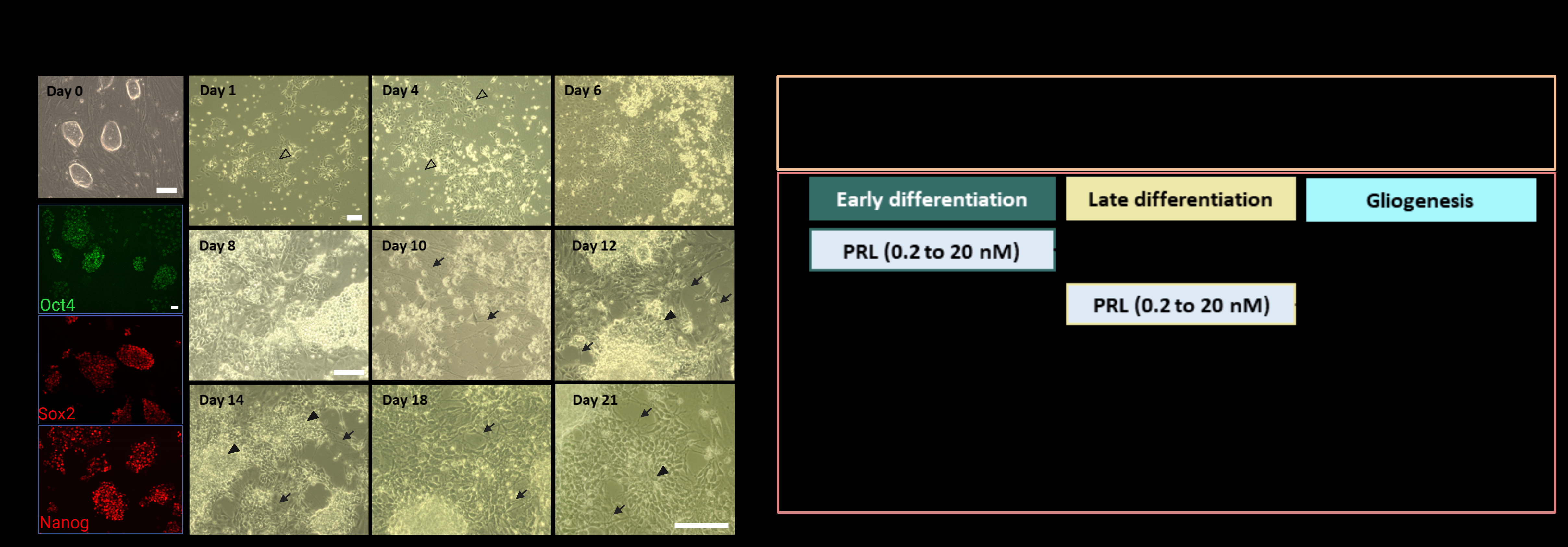

Supplement: Supplementary file 1 [file Image3.jpeg]

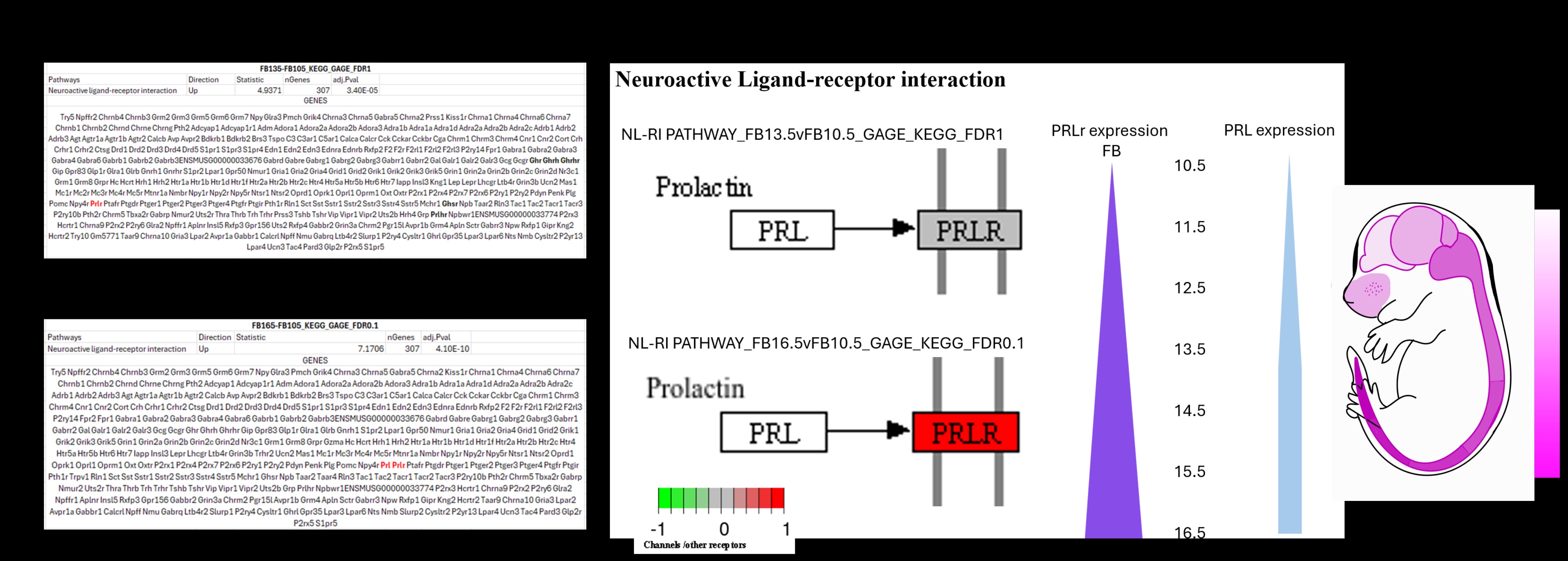

Supplement: Supplementary file 2 [file Image9.jpeg]

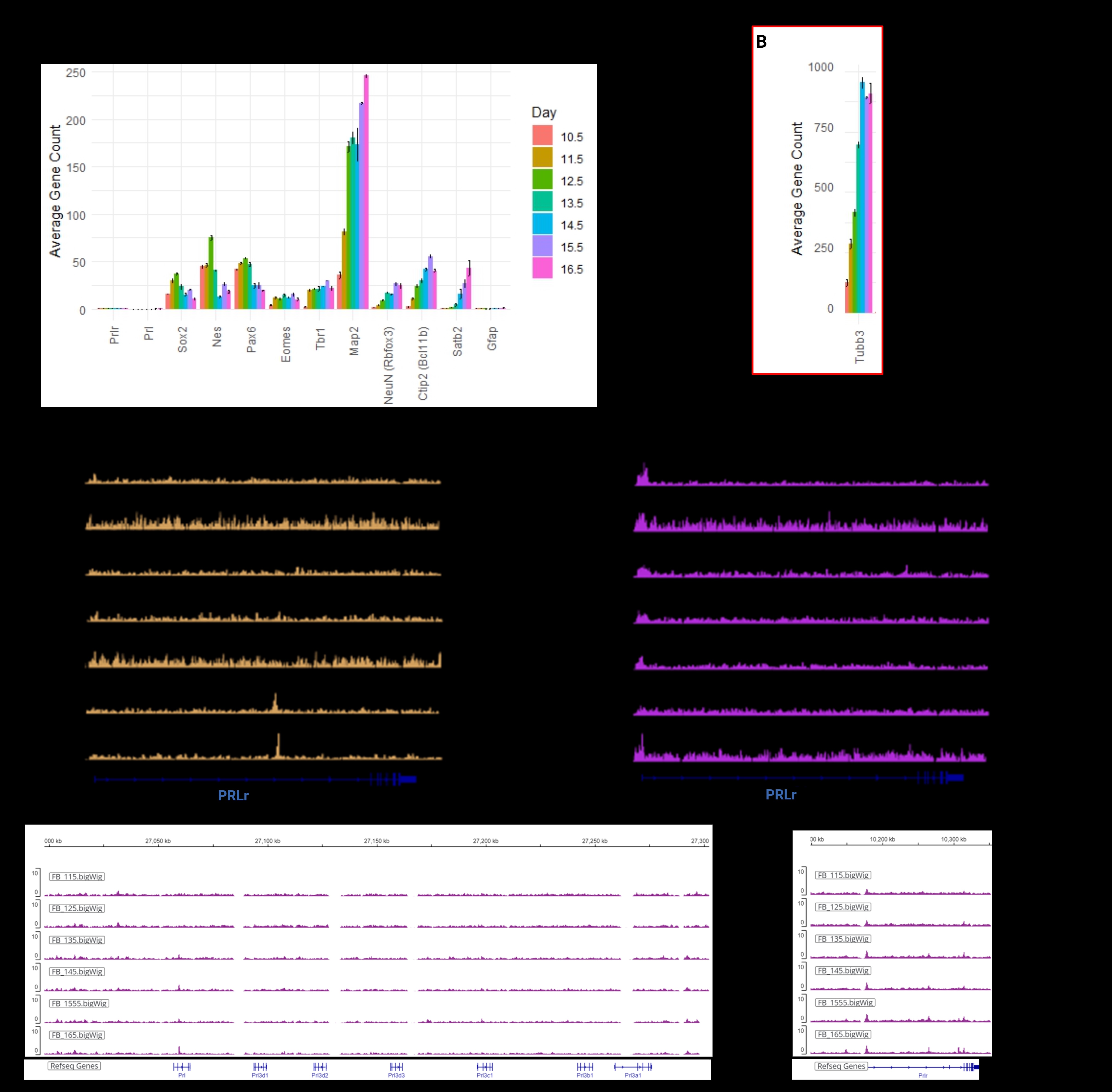

Supplement: Supplementary file 3 [file Image1.jpeg]

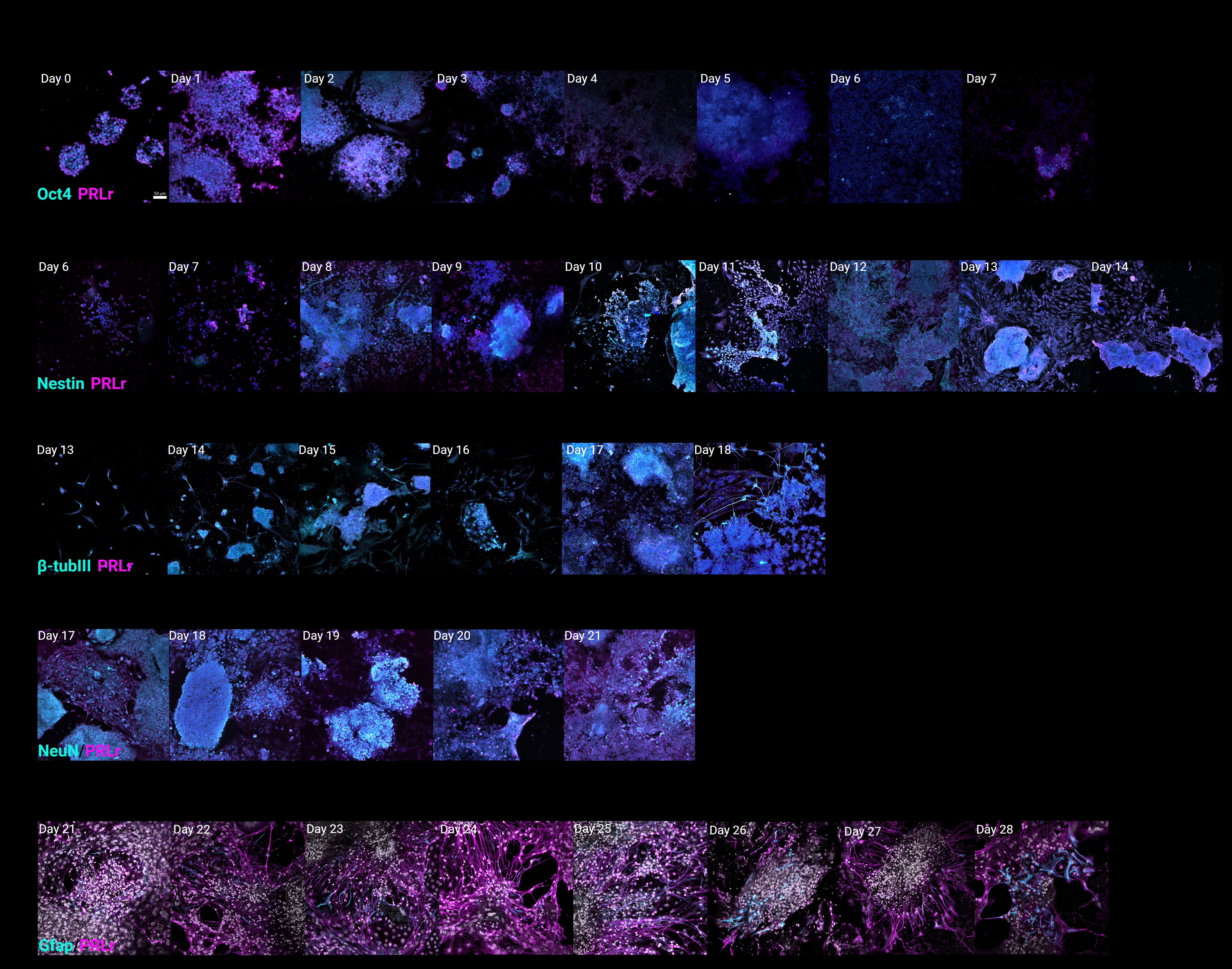

Supplement: Supplementary file 4 [file Image4.jpeg]

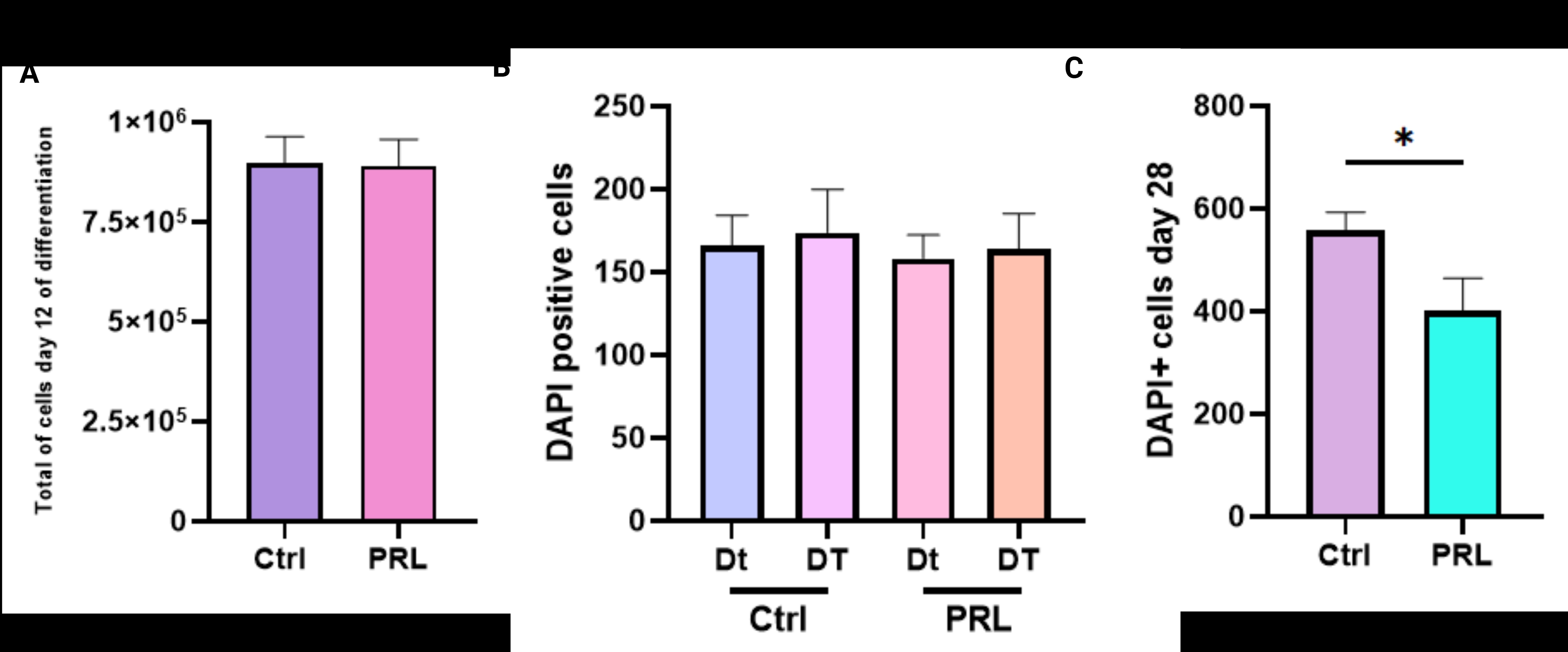

Supplement: Supplementary file 5 [file Image7.jpeg]

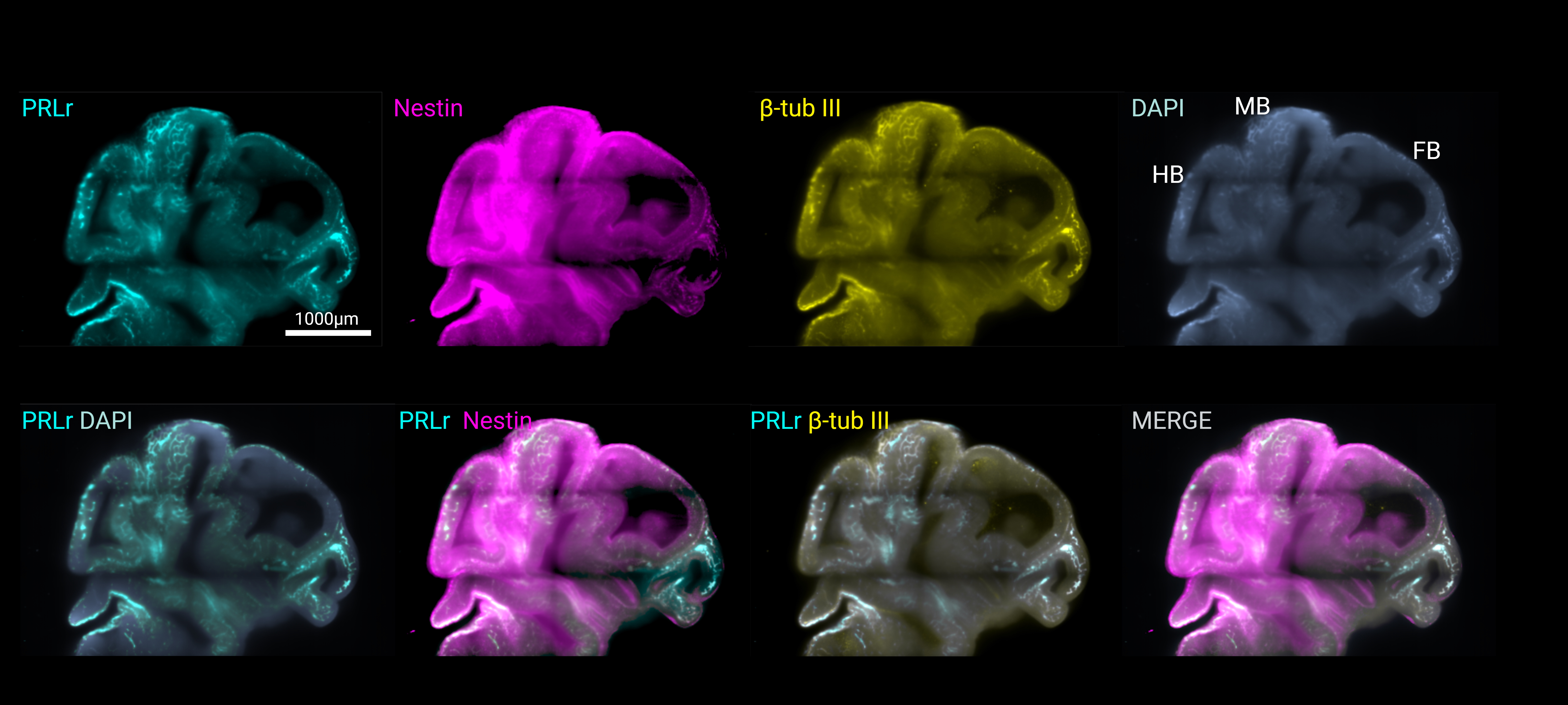

Supplement: Supplementary file 6 [file Image2.jpeg]

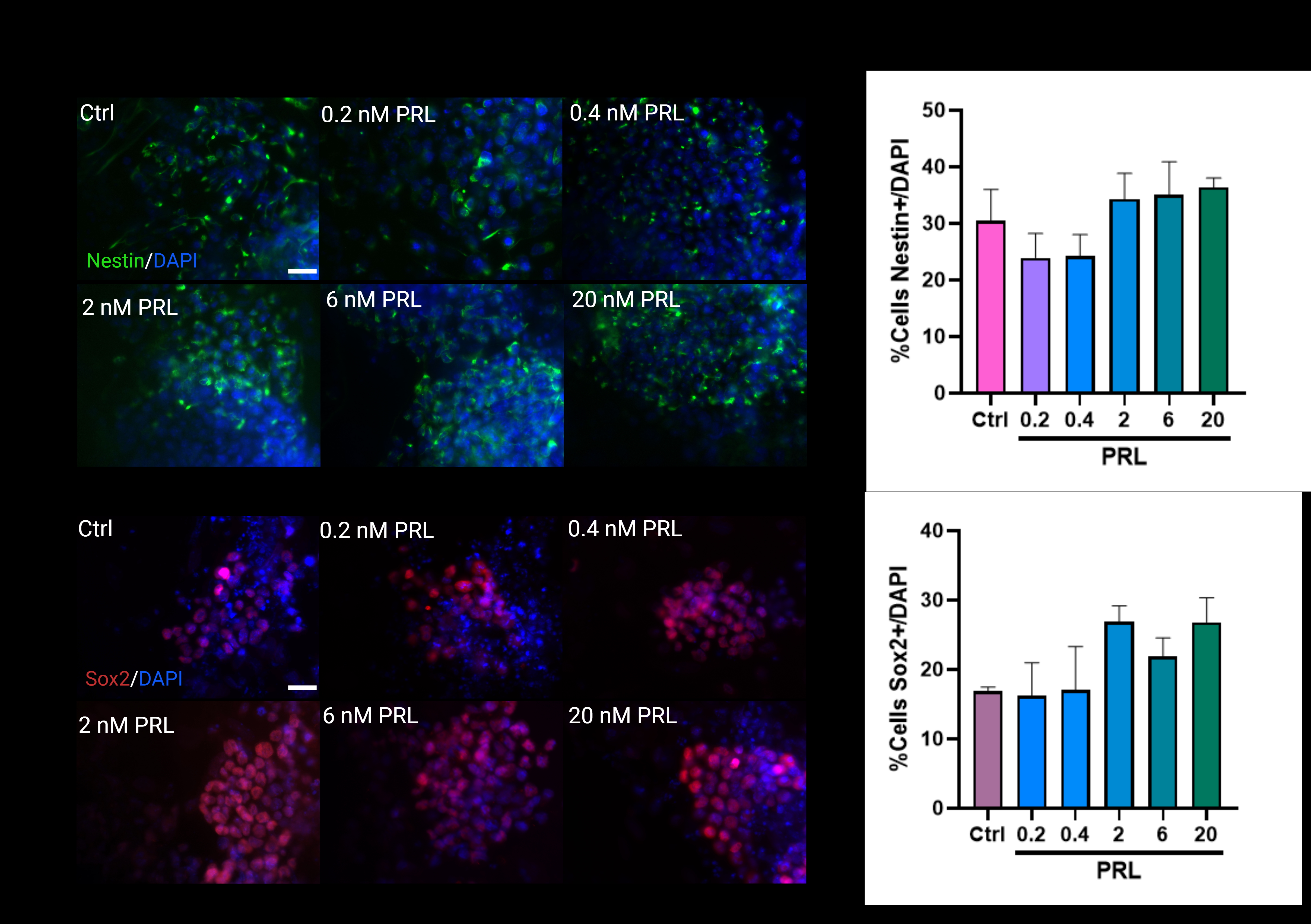

Supplement: Supplementary file 7 [file Image5.jpeg]

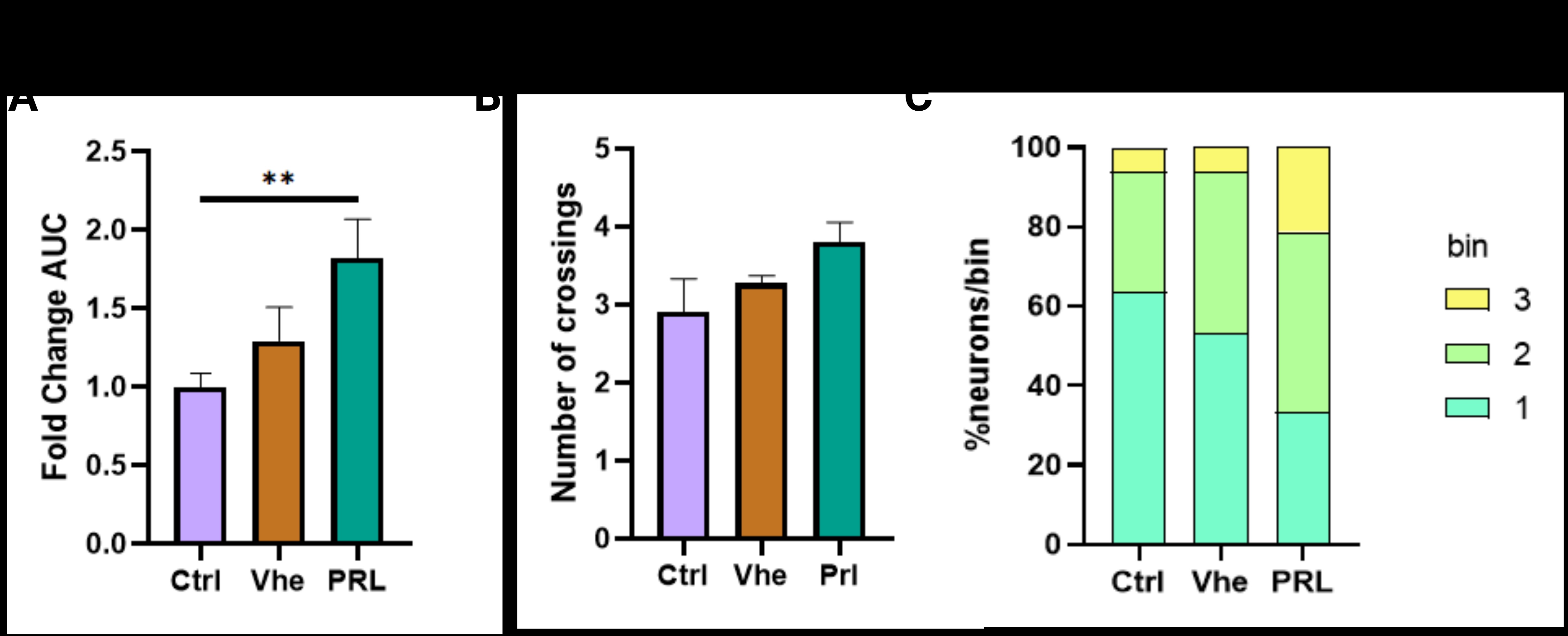

Supplement: Supplementary file 8 [file Image8.jpeg]

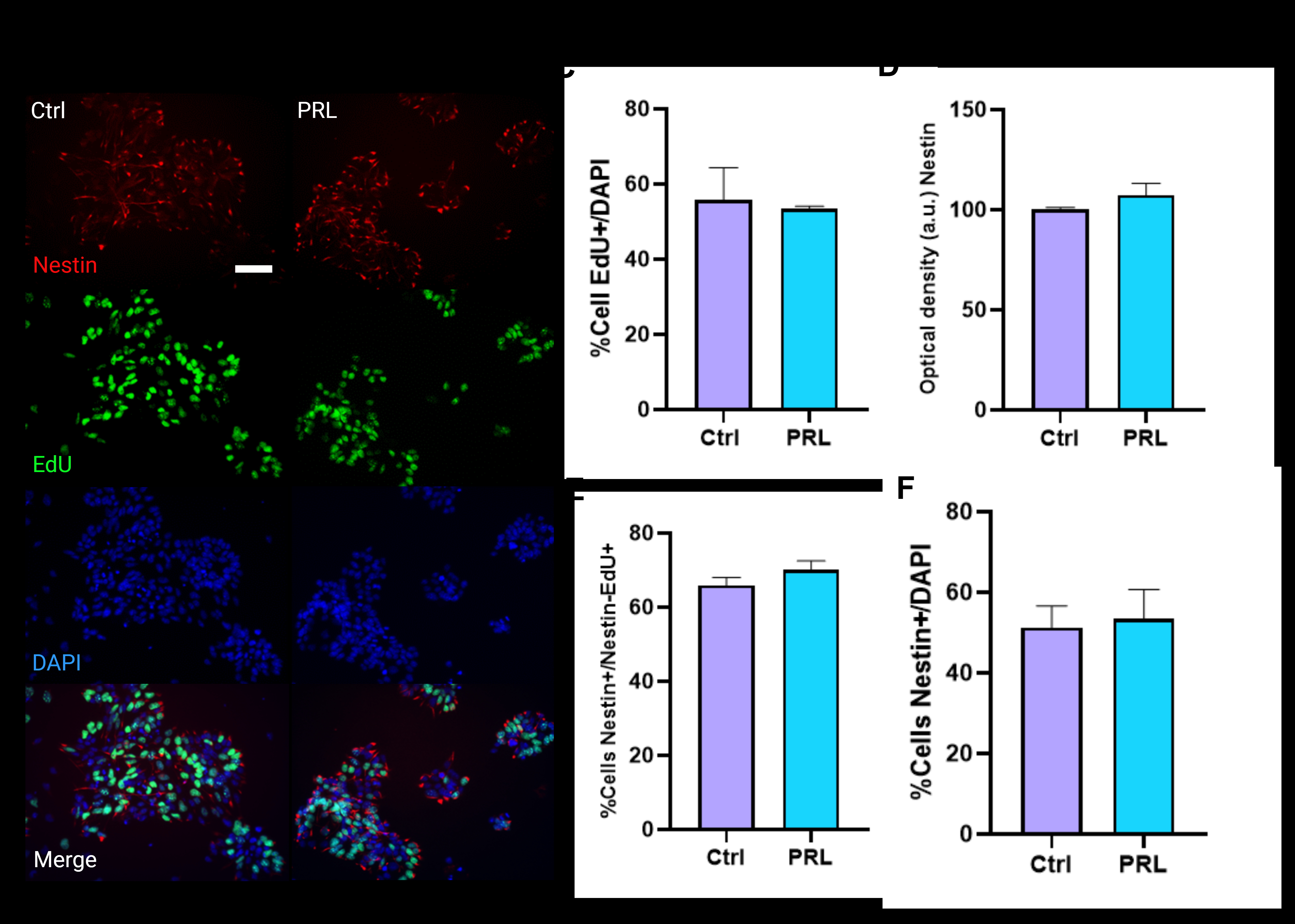

Supplement: Supplementary file 10 [file Image6.jpeg]
