# Supplementary material for: Prolactin drives cortical neuron maturation and dendritic development during murine embryonic stem cell differentiation
Source: Front Cell Dev Biol. 2025 Feb 26;13:1551090. doi: 10.3389/fcell.2025.1551090 (PMC11897521; doi:10.3389/fcell.2025.1551090)
Supplement: Supplementary file 9 [file DataSheet1.docx]

**Supplementary Table 1**. Comparison of neural phenotypes observed in this study with those reported in previous publications.

|  | % Positive cells | | |
| --- | --- | --- | --- |
|  | Martinez-Alarcon et al 202X | Gaspard, et al 2008 | Sadegh, et al 2014 |
| Sox2 | 16.88% (day 12) | N. d. | N. d. |
| Nestin | 30.42% (day 12) 55.73% (day 14) | 75% (day 14) | 48% (day 14) |
| β-tubullin III | 2.17% (day 12) | 15% (day 14) | 16% (day 14) |
| Tbr1 | 3.12% (day 21) | N. d. | 6.2% (day 21) |
| NeuN | 14.22% (day 21)  41.39% (day 28) | N. d. | 3.1% (day 21) |
| Map2 | 28.45% (day 21)  25.79% (day 28) | N. d. | N. d. |
| Gfap | 2.2% (day 21)  12.17(day 28) | 5% (day 21) | <1% (day 21) |

**Supplementary Table 2.** Primers used in this study.

| Name | Sequence | Product legth | Tm |
| --- | --- | --- | --- |
| PRLr | ACTTCTGACTGTGAGGACTTGCTGGT  AGGCCATGTTGTAGATTTGGATGTATC | 333 | 61.5°C |
| rRNA 18S | GGCCGTTCTTAGTTGGTGGA  CCCGGACATCTAAGGGCATC | 183 | 60°C |

**Supplementary Table 3.** Antibodies used in this study**.**

| Antibody | Clone ID | Host species | Reactivity | Source | Cat No. | Dilution |
| --- | --- | --- | --- | --- | --- | --- |
| Anti-Prolactin Receptor/PRL-R | EPR7184(2) | Rabbit | Mouse  Rat | Abcam | AB170935 | 1:100 |
| Anti-Oct3/4 | 40/Oct-3 (RUO) | Mouse | Human  Mouse | BD | 611202 | 1:100 |
| Anti-Sox2 |  | Rabbit | Human  Mouse | Abcam | AB5603 | 1:100 |
| Anti-Nanog |  | Rabbit | Mouse | Abcam | AB808892 | 1:100 |
| Anti-Nestin | Rat-401 | Mouse | Human  Mouse  Rat | Santa Cruz | SC-33677 | 1:200 |
| Anti- β-tubulin III/ Tuj1 | AB_10629222 | Chicken | Human  Mouse  Rat | Genetex | GTX85469 | 1:200 |
| Anti-TBR1 |  | Rabbit | Human  Mouse  Rat | Abcam | AB31940 | 1:200 |
| Anti-NeuN | 1B27 | Mouse | Human  Mouse  Rat | Abcam | AB104224 | 1:100 |
| Anti-Map2 |  | Rabbit | Human  Mouse | Genetex | GTX50810 | 1:100 |
| Anti-glial fibrillary acidic protein (Gfap) | G-A-5 | Mouse | Human  Rat  Pig | Sigma Aldrich | G3893 | 1:400 |
| Alexa flour 488  Goat-Anti-mouse |  | Goat | Mouse | Invitrogen | A11029 | 1:1000 |
| Alexa flour 568  Goat anti-Rabbit |  | Goat | Rabbit | Invitrogen | A11036 | 1:1000 |
| Alexa flour 488 Goat anti-Chicken |  | Goat | Chicken | Invitrogen | A110039 | 1:1000 |
| Alexa flour 647 Donkey anti- mouse |  | Donkey | Mouse | Invitrogen | A32728 | 1:1000 |
